# Supplementary material for: Changes in presentations with features potentially indicating cancer in primary care during the COVID-19 pandemic: a retrospective cohort study
Source: BMJ Open. 2021 May 24;11(5):e050131. doi: 10.1136/bmjopen-2021-050131 (PMC8154288; doi:10.1136/bmjopen-2021-050131)
Supplement: Supplementary data [file bmjopen-2021-050131supp001.pdf]

Supplementary File 1: Data Rules

1. Data extraction and analysis

- Data on patient characteristics, consultations and clinical codes for 21 practices were extracted by One Care.
- We only extracted consultations added by clinicians. Administrator added consultations (e.g. e-consultations and documents uploaded) were not extracted.
- Patient characteristics were extracted for all patients registered in July 2020.
- Consultations were extracted for all patients registered in July 2020.
- To calculate consultations per 1000 patients, we used the July 2020 patient list extracted by One Care as the denominator for the April-July 2020 consultations. For the April-July 2019 data, we adjusted this list size based on practice list size data (total list size of patients aged 50+ years for all 21 included practices) published by NHS digital. We calculated the quotient of July 2019/July 2020 list size, and used this as a multiplier for the denominator. We used this same method to individually calculate the offset for each practice for the negative binomial models. For the figures presenting data by month, we again used this same method but using monthly list sizes (for patients aged 50+ years) on NHS digital to calculate the multiplier for the denominator.

2. Consultation Type

We applied the following rules to identify consultation type:

Face-to-face: If

consultation category= "gp surgery" OR "face to face consultation" OR "face to face consultation with relative/carer" OR "emergency appointment" OR "extended hours consultation" OR "same day appointment" OR "urgent consultation".

Home: If
consultation category= "children's home visit note" OR "home visit note" OR "night visit note" OR "nursing home visit note" OR "residential home visit note" OR "twilight visit note"
OR
Clinical code text contains the words “home visit”.

Telephone: If
consultation category contains the words “telephone call” OR "telephone consultation” OR “enterprise consultation”[1]
OR
appointment type contains the words “telephone” OR “phonecall” OR “phone call” OR “telcon”
OR
appointment slot status= “telephone – complete”
OR

[1] These are consultations carried out in a GP surgery which is not the patients own surgery. We identified these as telephone consultation as we do not know anything more about the medium of these consultations and telephone was most likely. If clinical codes for video consultation were identified, this was coded to video instead, in accordance with the data rules shown in the following page.

Clinical code text contains the word “telephone” OR “telephone and/or video” OR “telephone/video” OR “telephone/ video” OR “telephone / video”

Video: If
consultation category= “consultation via video conference"
OR
Clinical code text= assessed by video consult" OR "consultation via video conference" OR "video consult"
E-consultation[2]: If
consultation category= “online communication” OR “consultation via multimedia” OR contains the word “e-mail”
OR
appointment type contains the words “e consult” OR “econsult” OR “e:consult”
OR
Clinical codes text contains the words “econsult” OR “e-consult” OR “e:consult” OR “e consult” OR “consultation via multimedia” OR “alert received from telehealth monitoring system”

We used the following rank order for determining consultation type: **Video, Telephone, E-consult, Home, Face-to-face** (E.g. if a patient has consultation type as “GP surgery” but has a clinical code for "video consult" then this is coded as video.)

Consultations were excluded as follows:

- Non consultation activity (e.g. where the consultation category was “administration note”, “inbound document”, and “scanned document”).
- Records which were appearing as consultations but were actually SMS messages. Identified as: *Consultation type*= ”Face-to-face” AND *consultation category*=”gp surgery” AND the consultation has a maximum of 3 clinical codes associated to it, and one is “patient mobile telephone number” or “patient telephone number”, and one is “sms (short message service) text message sent to patient” or “sms text message sent to patient” or “sms text sent to patient”
- Consultations which the patient did not attend, or did not answer the phone: Identified by (*Clinical code text* contains the words “did not attend” OR “failed encounter” OR “dna” AND *clinical code text* does not equal “dna studies” OR “dnacpr”) OR (*appointment slot status* = “telephone - not in” OR “dna” OR “walked out”).

3. Staff type

Codes were mapped for staff type as follows:

GP: Assistant GP, Associate practitioner - general practitioner, General medical practitioner, GP registrar, Locum GP, Salaried general practitioner, Sessional GP

[2] Because we only included consultations added by clinicians, not administrators, these only include e-consultations which clinicians added to the patient record. E-consultations added by administrators which covert to a telephone call (added by a GP) or dealt with by a task are not included.

Nurse/Paramedic: Advanced practitioner, Associate practitioner – nurse, Community nurse, Community practitioner, Enrolled nurse, Modern matron, Nurse consultant, Nurse manager, Paramedic, Paramedic specialist practitioner, Sister/charge nurse, Specialist practitioner, Staff nurse.

An Allied Professional Staff Type was also created. However, as the numbers of consultations were very small, this was not used in our analysis.

4. Ethnicity

Codes were mapped for ethnicity as follows:

- **White**: 44 different codes. Over 90% of patients were covered by the codes "White British", “British or mixed British” or "other white background."
- **Black/African/Caribbean/Black British**: 36 different codes. Over 90% were coded as “African”, “Caribbean”, “black African", “black Caribbean”, "Somali”, or “black British”.
- **Asian/Asian British**: 49 different codes. 90% of patients were covered by the codes “Indian or British Indian”, “other Asian background”, “Pakistani or British Pakistani", “Chinese”, “Bangladeshi or British Bangladeshi”, “Indian” or “Pakistani”.
- **Mixed/Multiple ethnic groups**: 27 different codes: 90% covered by “white and black Caribbean”, "other mixed background”, “white and Asian”, "white and black African”, “other mixed white”, “black and white”.
- **Other**: 40 codes including: “any other group”, “Turkish”, “Arab”, “other ethnic non-mixed (nmo)”, “Iranian”, "gypsy/Romany”, “Kurdish”, “Latin American”, “Albanian", “other European (nmo)”.
