# Supplementary material for: Changes in presentations with features potentially indicating cancer in primary care during the COVID-19 pandemic: a retrospective cohort study
Source: BMJ Open. 2021 May 24;11(5):e050131. doi: 10.1136/bmjopen-2021-050131 (PMC8154288; doi:10.1136/bmjopen-2021-050131)
Supplement: Author's manuscript [file bmjopen-2021-050131.draft_revisions.pdf]

BMJ Open is committed to open peer review. As part of this commitment we make the peer review history of every article we publish publicly available.

When an article is published we post the peer reviewers' comments and the authors' responses online. We also post the versions of the paper that were used during peer review. These are the versions that the peer review comments apply to.

The versions of the paper that follow are the versions that were submitted during the peer review process. They are not the versions of record or the final published versions. They should not be cited or distributed as the published version of this manuscript.

BMJ Open is an open access journal and the full, final, typeset and author-corrected version of record of the manuscript is available on our site with no access controls, subscription charges or pay-per-view fees (http://bmjopen.bmj.com).

If you have any questions on BMJ Open's open peer review process please email info.bmjopen@bmj.com

BMJ Open

Changes in presentations with features potentially indicating cancer in primary care during the COVID-19 pandemic: a retrospective cohort study

Journal:	*BMJ Open*
Manuscript ID	bmjopen-2021-050131
Article Type:	Original research
Date Submitted by the Author:	12-Feb-2021

Complete List of Authors:	Scott, Lauren; University Hospitals Bristol and Weston NHS Foundation Trust, National Institute for Health Research Applied Research Collaboration West; University of Bristol Medical School, Population Health Sciences<br>Murphy, Mairead; University of Bristol Faculty of Medicine and Dentistry, Centre for Academic Primary Care<br>Price, Sarah; University of Exeter Medical School,<br>Lewis, Rhys; One Care<br>Denholm, Rachel; University of Bristol, Centre for Academic Primary Care<br>Horwood, Jeremy; University of Bristol, UK, School of Social and Community Medicine<br>Salisbury, Chris; University of Bristol, Centre for Academic Primary Care

Keywords:	PRIMARY CARE, COVID-19, ONCOLOGY

SCHOLARONE™
Manuscripts

I, the Submitting Author has the right to grant and does grant on behalf of all authors of the Work (as defined in the below author licence), an exclusive licence and/or a non-exclusive licence for contributions from authors who are: i) UK Crown employees; ii) where BMJ has agreed a CC-BY licence shall apply, and/or iii) in accordance with the terms applicable for US Federal Government officers or employees acting as part of their official duties; on a worldwide, perpetual, irrevocable, royalty-free basis to BMJ Publishing Group Ltd ("BMJ") its licensees and where the relevant Journal is co-owned by BMJ to the co-owners of the Journal, to publish the Work in this journal and any other BMJ products and to exploit all rights, as set out in our licence.

The Submitting Author accepts and understands that any supply made under these terms is made by BMJ to the Submitting Author unless you are acting as an employee on behalf of your employer or a postgraduate student of an affiliated institution which is paying any applicable article publishing charge ("APC") for Open Access articles. Where the Submitting Author wishes to make the Work available on an Open Access basis (and intends to pay the relevant APC), the terms of reuse of such Open Access shall be governed by a Creative Commons licence – details of these licences and which Creative Commons licence will apply to this Work are set out in our licence referred to above.

Other than as permitted in any relevant BMJ Author's Self Archiving Policies, I confirm this Work has not been accepted for publication elsewhere, is not being considered for publication elsewhere and does not duplicate material already published. I confirm all authors consent to publication of this Work and authorise the granting of this licence.

For peer review only

Changes in presentations with features potentially indicating cancer in primary care during the COVID-19 pandemic: a retrospective cohort study

Lauren J Scott[1,2], Mairead Murphy[3], Sarah Price[4], Rhys Lewis[5], Rachel Denholm[3,6], Jeremy Horwood[1,3], Chris Salisbury[1,3]

[1] National Institute for Health Research Applied Research Collaboration West (NIHR ARC West), University Hospitals Bristol and Weston NHS Foundation Trust, Bristol, UK

[2] Population health Sciences, Bristol Medical School, University of Bristol, Bristol, UK

[3] Centre for Academic Primary Care (CAPC), Bristol Medical School, University of Bristol, Bristol, UK

[4] Exeter Medical School, University of Exeter, Exeter, UK

[5] One Care, Bristol, UK

[6] National Institute for Health Research Biomedical Research Centre, University of Bristol, Bristol, UK

For peer review only

Name	Qualification	Position	Institution	Email
Lauren J Scott	MSc	Senior Research Associate	University of Bristol	Lauren.Scott@bristol.ac.uk
Mairead Murphy	PhD	Research Fellow	University of Bristol	Mairead.murphy@bristol.ac.uk
Sarah Price	PhD	Research Fellow	University of Exeter	S.J.Price@exeter.ac.uk
Rhys Lewis	MPH	Senior Business Analyst	One Care	Rhys.lewis@onecare.org.uk
Rachel Denholm	PhD	Lecturer	University of Bristol	R.denholm@bristol.ac.uk
Jeremy Horwood	PhD	Associate Professor	University of Bristol	J.horwood@bristol.ac.uk
Chris Salisbury	MD, FRCGP	Professor	University of Bristol	C.salisbury@bristol.ac.uk

Corresponding author:
Mairead.murphy@bristol.ac.uk
Centre for Academic Primary Care
Bristol Medical School
Bristol BS8 2 PS

Word count=3,317.

For peer review only - http://bmjopen.bmj.com/site/about/guidelines.xhtml

UOB Confidential & Sensitive

Abstract

Objectives

To investigate how the COVID-19 pandemic affected the number of people aged 50+ presenting to primary care with features which could potentially indicate cancer, and to explore how reporting differed by patient characteristics and in face-to-face vs. remote consultations.

Design, Setting and Participants

A retrospective cohort study of GP, nurse, and paramedic primary-care consultations in 21 practices in South-West England.

Main outcome measures

Potential indicators of cancer were identified using code lists for symptoms, signs, test results and diagnoses listed in the National Institute for Health and Care Excellence (NICE) suspected-cancer referral guidance (NG12).

Results

During April-July 2019 and 2020, respectively, 69,254/123,947 (56%) and 56,314/126,466 (45%) registered patients aged 50+ years consulted, with 17% and 11% respectively reporting a potential cancer indicator (incidence rate ratio [IRR]=0.64, 95% confidence interval [CI] 0.62 to 0.6, p<0.001). Reductions in potential cancer indicators were stable across age group, sex, ethnicity, index of multiple deprivation (IMD) quintile, and shielding status, but less marked in patients with mental health conditions than without (IRR=0.75, 95% CI 0.72 to 0.79, interaction p<0.001). Proportions of GP consultations with potential indicators of cancer reduced between 2019 and 2020 for face-to-face consultations (IRR=0.84, 95% CI 0.76 to 0.92, p<0.001) and increased for remote consultations (IRR=1.17, 95% CI 1.07 to 1.29, p=0.001), though it remained lower in remote consulting than face-to-face in April-July 2020. This difference was greater for nurse/paramedic consultations (face-to-face: IRR=0.61, 95% CI 0.44 to 0.83, p=0.002; remote: IRR=1.60, 95% CI 1.10 to 2.333, p=0.014).

Conclusion

Patients consulting with presentations that could potentially indicate cancer reduced during the first-wave of the COVID-19 pandemic. Patients should be encouraged to continue contacting primary care for persistent signs and symptoms, and GPs and nurses should be encouraged to probe patients for further information during remote consulting, in the absence of non-verbal cues.

Key words: COVID-19, General Practice, Cancer

Strengths and limitations of this study

- We were able to use a large number of patients (>126,000) aged 50+ years from a diverse range of backgrounds, and a comprehensive list of potential cancer indicators.
- We were able to identify the number of patients presenting to primary care with symptoms, signs, test results or diagnoses which could potentially indicate cancer, and to explore how this reporting differed by patient characteristics and consultation provision.
- We have distinct and clear findings; that the proportion of patients presenting with symptoms, signs, test results or diagnoses which could indicate cancer reduced during the COVID-19 pandemic and that reporting of potential cancer indicators increased in remote consultations and decreased in face-to-face consultations during the pandemic, compared to 2019.
- We were unable to take account of whether symptoms were persistent or unexplained, which may inflate our estimates of potential cancer indicators.
- Some symptoms and signs may have been reported in free text rather than using clinical codes or may have been recorded in administration notes not associated with consultations.

For peer review only - http://bmjopen.bmj.com/site/about/guidelines.xhtml

UOB Confidential & Sensitive

Introduction

In March 2020, the World Health Organisation declared COVID-19 a pandemic[1], and the UK went into lockdown; the public were instructed to "Stay at home, protect the NHS, and save lives".[2] Primary care providers were advised to provide all consultations remotely, unless a face-to-face consultation was urgently required,[3] in order to reduce contact and the spread of the virus. Patients at particularly high risk of severe COVID-19 due to age or pre-existing health conditions were advised to 'shield' and avoid all but essential contact.

GP and nurse consulting rates markedly dropped in April-May 2020 during the period of UK lockdown, but had largely returned to normal by July 2020,[4] with the majority of consultations carried out remotely (mostly via telephone).

There are fears that reduced consulting during the lockdown period and changes in consultation provision may have adversely impacted on cancer detection.[5] There was a 63% reduction in skin cancer diagnosis in England in the first three month of the pandemic[6] and 3500 fewer colorectal cancer diagnoses than expected in the first six months.[7] This pattern is not limited to the UK. An analysis on the Danish registry showed cancer diagnosis in Denmark dropped by 33% from March to May 2020[8] and similar reductions have been observed in the Netherlands.[9]

These concerning reductions in diagnoses are likely to result from a combination of factors, including temporary suspensions in cancer screening services,[10 11] reduced capacity in secondary care and less reporting of potential cancer symptoms in primary care. A Cancer Research UK survey suggested GPs perceived they received fewer reports of cancer symptoms, particularly from older people, after the pandemic than before.[5] This could be related to changes in the willingness of patients to disclose symptoms, or changes in patient or doctor behaviour associated with the shift to remote consultations. Reduced symptom reporting could result in late diagnoses, increased workload for cancer services, and poorer patient outcomes.[12] A modelling study showed that delays in cancer diagnosis in this period could result in approximately 3,330 deaths for breast, colorectal, lung and oesophageal cancer alone.[13]

Aims: The aim of this study was to investigate how the COVID-19 pandemic affected the number of people aged 50 years and over presenting to primary care with symptoms, signs, test results or diagnoses which could potentially indicate cancer, and to explore how this reporting differed by patient characteristics and consultation provision (i.e. face-to-face vs. remote).

Methods

Design and setting
This was a retrospective cohort study of primary care consultations in 21 practices in the South West of England. Data were extracted for the RAPCI Study (Rapid COVID-19 intelligence to improve primary care response), a mixed-methods study on how general practices coped with rapid change to remote consulting in the initial months of the COVID-19 pandemic. The main results are reported elsewhere.[4]

Data
Routinely collected and anonymised data were provided by One Care, the GP federation in Bristol, North Somerset, and South Gloucester Clinical Commissioning Group. All practices use the same EMIS electronic medical records system. All patients registered in July 2020 were included. Patient data included demographics (age, sex, ethnicity, and deprivation) and clinical characteristics (such as

For peer review only - http://bmjopen.bmj.com/site/about/guidelines.xhtml
UOB Confidential & Sensitive

mental health and shielding status). Data was obtained on all consultations added to the system by clinical staff, and all clinical codes added during those consultations between February 2019 and July 2020 inclusive. For the analyses, the period April-July 2020 (i.e. the period since the start of UK lockdown) was compared to the same period the previous year (April-July 2019). For this paper, we restricted the analysis to patients aged 50 years and over as cancer is relatively rare in younger age groups.

Consultations

A consultation was defined as an interaction between a patient and a GP, nurse, or paramedic working in general practice. We excluded consultations recorded by administrators or other health care professionals, and administrative tasks recorded by clinicians. Remote consultations were defined as those carried out by telephone, video, or e-consultation. Face-to-face consultations were those carried out in GP practices as well as visits to patients' homes or nursing homes. See supplemental material 1 for further details.

Outcomes

Pre-existing code lists[14 15] were used to identify records of potential cancer indicators associated with a consultation. The indicators were collated from the clinical features of undiagnosed cancer (symptoms, signs, abnormal test results or diagnoses) listed in the National Institute for Health and Care Excellence guidance on the recognition and referral of suspected cancer (NG12)[16], using robust methods.[15] Lists were developed using Read codes and subsequently mapped to SNOMED CT codes using medical dictionaries provided by the Clinical Practice Research Datalink for its AURUM dataset.[17] SNOMED CT codes were used for this analysis. We excluded the indicator for new-onset diabetes (defined as a diabetes code in the absence of any diabetes monitoring in the previous 2 years), because we did not have the necessary patient records required to calculate this.

A binary variable for 'any potential cancer indicators' was derived based on whether a consultation contained at least one potential cancer indicator code (Yes) or did not (No). Using this consultation data, the number of patients reporting potential cancer indicators was calculated.

Each potential cancer indicator was categorised based on how many patients reported it in April-July 2019: most commonly (≥0.5%), less commonly (0.1% to <0.5%), rarely (0.02% to <0.1%), and very rarely reported (<0.02%). We felt it was important to separate these, as the most commonly reported indicators include symptoms which often indicate acute illness or chronic illnesses other than cancer (e.g. cough), whereas the less commonly/rarely reported indicators are more likely to be associated with cancer (e.g. weight loss, lumps and masses).

Explanatory variables

Age (in July 2020) was split into four categories: 50-59 years, 60-69 years, 70-79 years, and 80+ years old. Deprivation quintiles (measured by the index of multiple deprivation score; IMD) were calculated using the IMD deciles recorded in the patient record, which are based on Lower Super Output Areas of residence. Ethnicity was derived by mapping ethnicity descriptions in primary care records to one of five ethnicity categories: white, Asian, Black, mixed, and other (see supplemental material 1). Presence of a mental health condition was defined as either severe mental illness (defined according to the Quality and Outcomes Framework rules[18]), diagnosed depression, or prescribed anti-depressants (excluding tricyclics, commonly used for non-mental health related conditions) in the three months prior to July 2020. Sex and shielding status (as of July 2020) were obtained directly from patients' primary care records.

For peer review only - http://bmjopen.bmj.com/site/about/guidelines.xhtml

UOB Confidential & Sensitive

Statistical analysis

The number and percentage of patients reporting potential cancer indicators during our reporting periods are presented. In addition, consultation rates (and percentages with potential cancer indicators) are reported per 1,000 registered patients. Practice list sizes were based on July 2020 data, and adjusted to account for historic list sizes using NHS digital data[19] ('adjusted list size'; see supplemental material 1).

Changes in proportions of patients presenting (per practice) with any potential cancer indicator in April-July 2020 compared to April-July 2019 were investigated using negative binomial regression models with counts of patients presenting with potential cancer indicators as the outcome; incidence rate ratios (IRRs) and 95% confidence intervals (CIs) are reported. Consultation year was fitted as a fixed effect, GP practice as a random effect, and adjusted practice list size (per level of covariate where appropriate) as the offset. Fixed effects for each of the categorical patient characteristics of interest (age, sex, ethnicity, IMD quintile, mental health status, and shielding status), along with an interaction term between each of these covariates and consultation year, were separately fitted to the model; interaction p-values are presented and changes in proportions of patients with potential cancer indicators are only presented separately for each level of a covariate if the interaction p-value was <0.05. Model validity was checked using standard methods; outliers which disrupted model fit were removed.

To investigate the effect of consultation provision (i.e. face-to-face vs. remote consulting), we looked at proportions of consultations with potential cancer indicators (out of all consultations) rather than proportions of patients. Separate models were fitted for consultations with GPs and nurses/paramedics. Models were similar to those described above, with number of consultations as the outcome, a fixed effect for consultation provision and an interaction with year, GP practice as a random effect, and total numbers of consultations per practice per level of consultation provision as the offset.

For individual potential cancer indicators, unadjusted IRRs comparing the percentage of patients consulting in April-July 2020 to 2019 are presented to help interpretation, but due to the large number of indicators and resultant issues with multiple testing, no modelling was performed.

Stata 15.1 was used to conduct all data checking, cleaning, and analyses.

Patient and Public involvement (PPI)

A PPI group reviewed the RAPCI proposal before submission, agreed with the importance of the study and commented on the plain language summary. A meeting was also held to discuss preliminary findings of the main study[4], where a number of points were raised which supported the current work. These included participants highlighting that they felt things were missed during their telephone consultations because the GP could not see them.

Results

During April-July 2019, of the 123,947 registered patients over 50, 69,254 (56%) consulted, and 21,252 (17%) of patients reported a potential cancer indicator. In April-July 2020, of 126,466 registered patients over 50, 56,314 (45%) consulted and 13,829 (11%) reported an indicator. This comprised 1837 consultations per 1000 patients registered (with indicators reported in 16% of consultations) in April-July 2019 and 1663 consultations per 1000 patients (with indicators reported in 13% of consultations) in April-July 2020. Figures 1 and 2 demonstrate that patients consulting, and consultation rates, substantially dropped in April 2020 (following UK lockdown), and while

For peer review only - http://bmjopen.bmj.com/site/about/guidelines.xhtml

UOB Confidential & Sensitive

consultation rates had recovered by July 2020, the proportion of patients who consulted had not. Further, the percentage of patients reporting potential cancer indicators, and percentage of consultations with indicators reported, also dropped in April 2020; both had increased by July 2020, but neither had recovered to pre-COVID levels. Patients who consulted in 2020 were of similar age, sex, IMD quintile and ethnicity as those consulting in 2019, but may have been more likely to have a mental health condition or be shielding. (Table 1).

There was a 36% reduction in patients who reported potential cancer indicators in April-July 2020 compared to 2019 (IRR 0.64, 95% CI 0.62 to 0.67, p<0.001; Table 2). This reduction was stable across age group (interaction p-value=0.136), sex (p=0.467), ethnicity (p=0.471), IMD quintile (p=0.585), and shielding status (p=0.099), but differed by mental health status (p<0.001; Table 2). The reduction in reporting of these indicators was less pronounced in patients with a mental health condition (IRR 0.73, 95% CI 0.69 to 0.77, p<0.001) than patients without a mental health condition (IRR 0.63, 95% CI 0.61 to 0.66, p<0.001; Table 2).

The proportion of consultations where potential cancer indicators were reported differed by consultation provision for both GP and nurse/paramedic consultations (both p<0.001; Table 3). In GP consultations, proportions of face-to-face consultations with indicators reported dropped between 2019 and 2020 (IRR 0.84, 95% CI 0.76 to 0.92, p=<0.001) and increased for remote consultations (IRR 1.17, 95% CI 1.07 to 1.29, p=0.001; Table 3). For nurse/paramedic consultations, this finding was even more extreme (IRR 0.61, 95% CI 0.44 to 0.83, p=0.002 for face-to-face consultations; IRR 1.60, 95% CI 1.10 to 2.33, p=0.014 for remote consultations). Despite this increase in the proportion of potential cancer indicators in remote consultations, in both years GP remote consultations were less likely to report potential cancer indicators compared with GP face-to-face consultations (Table 3).

The proportion of patients reporting all commonly reported indicators reduced between the two periods (unadjusted IRR=0.59), with the biggest reductions in chest infections (IRR=0.25), fever (IRR=0.36), coughs (IRR=0.44) and fatigue (IRR=0.50; Table 4). For less commonly reported and rarely reported indicators, the reductions were less marked (IRR=0.75 and 0.76, respectively). Erectile dysfunction (IRR=0.40), irritable bowel syndrome (IRR=0.49), limp/gait abnormalities (IRR=0.39), night sweats (IRR=0.43) and lymphadenopathy (IRR=0.43) saw the largest reductions in these groups. Less common indicators which were similar or even increased were: nausea (IRR=1.00), weight loss (IRR=1.07) and constipation (IRR=1.18).

Discussion

Summary of findings

In April 2020, coinciding with UK lockdown, both the proportion of patients consulting, and those presenting with features potentially indicating cancer reduced compared to April 2019. By July 2020, although overall consultation rates had returned to previous levels, the proportion of patients consulting, and the proportion with potential cancer indicators remained lower than the previous year. The reduction in potential cancer indicator reporting did not differ by age, sex ethnicity, deprivation, or shielding status, but was less evident in patients with mental health conditions than those with no mental health conditions. Our results show that, despite an increase in consultations (for any reason) in patients aged 85+ and patients advised to shield (shown in our previous paper[4]), these have not resulted in higher levels of potential cancer indicator reporting in these groups, perhaps indicating these consultations were for routine assessment rather than to address new issues. Patients with mental health conditions did, however, have a smaller reduction in presentations with features potentially indicating cancer than other patients, perhaps as a result of

For peer review only - http://bmjopen.bmj.com/site/about/guidelines.xhtml

UOB Confidential & Sensitive

this increased focus on them from GPs.[4] Between 2019 and 2020, there was a large reduction in face-to-face consultations which involved reporting of cancer indicators, which was not fully compensated for by an increase in reporting of these indicators in remote consultations. Of the most commonly reported indicators, chest infections, fever, coughs and fatigue reduced most dramatically.

Strengths and limitations

To our knowledge, this is the first paper to assess the effect of the COVID-19 pandemic on potential cancer indicator reporting in UK primary care. The analysis is based on a comprehensive list of potential cancer indicators, developed and validated by Price, which has been used in previous research and is available on request.[14 15] Our analysis included a large number of patients (>126,000) aged 50+ years from a diverse range of backgrounds, and as changes in service provision due to the pandemic have affected the whole country, our findings are likely to be generalisable to other areas of England. Except for ethnicity, missing data about patient characteristics were low. There are several limitations which pertain to the recording of potential cancer indicators. Firstly, some symptoms and signs may have been reported in free text rather than using clinical codes,[20] or may have been recorded in administration notes not associated with consultations. Further, there were a small number of Read codes in the pre-defined list which do not have equivalent SNOMED CT codes, although the frequency with which these codes were used was so low as to be materially insignificant. These issues may have resulted in systematic under-reporting of potential cancer indicators; however, this is likely to be similar in both years and therefore should not affect the comparative findings. A further limitation was that new SNOMED codes added since the changeover from Read codes to SNOMED did not appear in our pre-defined lists, an issue which would affect April-July 2020 but not 2019; however, we checked this for common indicators cough and back pain, and found new codes were used very rarely (data not shown). Finally, for indicators to be of concern as possible harbingers of cancer, they are sometimes qualified by age at onset, or as persistent or unexplained in National Institute for Health and Care Excellence suspected-cancer guidance (NG12). Therefore, we limited our analysis to patients over 50 years of age (because cancer is more common in older patients) but were unable to take account of whether symptoms were persistent or unexplained. This limitation applies to other studies based on routine records and using similar methods[14], but the effect may be to inflate our estimates of potential cancer indicators.

Comparison with other literature

Few other studies have looked at the content of primary care consultations following UK lockdown during the COVID 19 pandemic. Analysis of primary care data from a deprived urban population found that diagnoses of common conditions decreased substantially between March and May 2020, suggesting a large number of patients may have undiagnosed conditions resulting from changes in access post-lockdown.[21] Our study adds to these findings for cancer-specific diagnoses.

Cancer screening programmes, estimated to account for about 5% of cancer diagnoses, were suspended in the wake of the pandemic.[10] This made symptom-based diagnosis all the more urgent. UK lockdown could have affected symptom-based reporting via the move to remote consulting. Research has shown that telephone and video consultations result in fewer problems being identified than face-to-face consultations.[22] Lockdown could also have influenced patients to only contact primary care if they thought their problem was serious, which is likely to be reflected in the reduced proportion of patients consulting found in our study.

For peer review only - http://bmjopen.bmj.com/site/about/guidelines.xhtml

UOB Confidential & Sensitive

Early in the pandemic, GPs predicted that patients with well-recognised red-flag symptoms, such as a new lump or rectal bleeding, would continue to present to primary care, but that vaguer cancer symptoms such as fatigue, change in bowel habit, and weight loss might be dismissed by the patient as trivial and not presented to primary care.[10] This was supported by our findings, which show that the most common symptoms reduced more substantially than less common ones. Of course, many of these more common symptoms (such as chest infections and sore throats) will be attributable to causes other than cancer, such as viral infections, and the figures should be interpreted in this context. This is supported by the fact that data from the Royal College of General Practitioners research and surveillance centre showed a marked reduction in weekly reported incidence of asthma, intestinal infectious diseases, and upper and acute respiratory tract infections from week 12 of 2020.[23] Nonetheless, the reductions are still concerning, especially for older patients. A 2020 survey from Cancer Research UK suggested GPs are particularly worried about older people not consulting with potential cancer symptoms compared to before the pandemic.[5] Analysis of the national cancer registry in the Netherlands shows reduction in cancer diagnosis at the start of the pandemic was particularly pronounced in the over 80s.[9] The Netherlands operates a similar GP gatekeeper model to the UK[24], and the authors of this Dutch paper suggested that the move to remote GP consulting combined with patients delaying consultation about potential non-specific cancer symptoms may have contributed to the reduction in diagnoses.[9] In the current study we show that this suggestion is supported for the set of UK practices we analysed. Although we did not observe a greater reduction in symptom reporting in the over 80s, potential symptoms in over 80s are more likely to result in a cancer diagnosis; therefore, the fact that reductions were similar across all age groups, rather than less of a reduction seen in older age groups, is of concern.

Implications for policy and practice

Combined with our previous work,[4] the Cancer Research UK report,[5] and reductions in cancer diagnoses[6 7] these findings suggest that patients are less likely to report potential cancer indicators than before the COVID-19 pandemic, particularly for more common symptoms such as fever and coughs. In the context of repeated lockdowns, it is therefore extremely important that the general public are advised that they should still consult with primary care if they have persistent symptoms that might indicate cancer. Further, GPs and nurses should be encouraged to ask more probing questions during remote consulting, as they may miss symptoms which may have previously been picked up from non-verbal cues and possibly a more open discussion face-to-face.

Conclusion

The proportion of patients consulting with primary care, as well as those reporting potential cancer indicators, reduced during the first-wave of the COVID-19 pandemic. While there has been an increased rate of potential cancer indicators reported in remote consultations compared to last year, there are still lower rates in remote GP consultations than in face-to-face, suggesting remote consulting may be part of the reason for the reduction in reporting of potential cancer indicators.

For peer review only - http://bmjopen.bmj.com/site/about/guidelines.xhtml

UOB Confidential & Sensitive

Acknowledgements: The authors would like to thank all the participants in this study, Bristol North Somerset and South Gloucestershire Clinical Commissioning Group, One Care for providing the data extract, the NIHR Clinical Research Network for adopting the study on the NIHR portfolio, and the NIHR SPCR for funding the research. We would also like to thank Willie Hamilton for his work on developing the potential cancer indicator lists (alongside author Sarah Price).

Funding: This study was funded by the National Institute for Health Research (NIHR) School for Primary Care Research. Additional funding for staff time was provided by the Applied Research Collaboration West (ARC West) at University Hospitals Bristol and Weston NHS Foundation Trust and One Care. Professor Salisbury is a National Institute for Health Research (NIHR) Senior Investigator. The funder had no role in the study design, collection and analysis of data, or the writing of the manuscript. The views expressed are those of the authors and not necessarily those of, the NIHR or the Department of Health and Social Care.

Ethical approval: This study received ethical approval from the University of Bristol Faculty of Health Sciences Research Ethics Committee (ID 103166), and Health Research Authority approval (IRAS project ID 282541; REC reference 20/HRA/2070). The study was sponsored by the University of Bristol.

Competing interests: The authors declare that they have no competing interests

Data sharing: Potential cancer indicator codes lists are available from the authors on request.

Transparency statement: All authors affirm that the manuscript is an honest, accurate, and transparent account of the study being reported, that no important aspects of the study have been omitted, and that any discrepancies from the study as originally planned have been explained.

Dissemination declaration: Results will not be disseminated to study participants as they were not directly involved or consented into the study and it would therefore not be appropriate.

Contributions of authors: LS, MM, RD, JH, and CS contributed to the conception and design of the study, SP provided the potential cancer indicator code lists and interpretation of these, RL extracted the data, MM led the main study, and LS performed the analysis and drafted the manuscript. All authors contributed to the organisation and conduct of the study, the interpretation of study data and results, and critiqued the manuscript for important intellectual content.

For peer review only

For peer review only - http://bmjopen.bmj.com/site/about/guidelines.xhtml

UOB Confidential & Sensitive

References

1. Organisation WH. WHO Director-General's opening remarks at the media briefing on COVID-19 - 11 March 2020, 2020.
2. Care DoHaS. Coronavirus: stay at home, protect the NHS, save lives - web version 2020 [Available from: https://www.gov.uk/government/publications/coronavirus-covid-19-information-leaflet/coronavirus-stay-at-home-protect-the-nhs-save-lives-web-version.
3. Andrea D. GPs urged to go digital to prevent spread of coronavirus 2020 [updated 2020/10/03/. Available from: https://www.digitalhealth.net/2020/03/gps-urged-to-go-digital-to-prevent-spread-of-coronavirus/.
4. Murphy M, Scott LJ, Salisbury C, et al. The implementation of remote consulting in UK primary care following the COVID-19 pandemic: a mixed-methods longitudinal study. *Research Square- PRE-PRINT* 2020 doi: 10.21203/rs.3.rs-94227/v1
5. Cancer Research UK. GPs say elderly are not seeking help for potential cancer symptoms, 2020.
6. Andrew TW, Alrawi M, Lovat P. Reduction in skin cancer diagnoses in the UK during the COVID-19 pandemic. *Clin Exp Dermatol* 2021;46(1):145-46. doi: 10.1111/ced.14411 [published Online First: 2020/08/06]
7. Morris EJA, Goldacre R, Spata E, et al. Impact of the COVID-19 pandemic on the detection and management of colorectal cancer in England: a population-based study. *Lancet Gastroenterol Hepatol* 2021 doi: 10.1016/S2468-1253(21)00005-4 [published Online First: 2021/01/18]
8. Skovlund CW, Friis S, Dehlendorff C, et al. Hidden morbidities: drop in cancer diagnoses during the COVID-19 pandemic in Denmark. *Acta Oncol* 2021;60(1):20-23. doi: 10.1080/0284186X.2020.1858235 [published Online First: 2020/12/09]
9. Dinmohamed AG, Visser O, Verhoeven RHA, et al. Fewer cancer diagnoses during the COVID-19 epidemic in the Netherlands. *Lancet Oncol* 2020;21(6):750-51. doi: 10.1016/S1470-2045(20)30265-5 [published Online First: 2020/05/04]
10. Jones D, Neal RD, Duffy SRG, et al. Impact of the COVID-19 pandemic on the symptomatic diagnosis of cancer: the view from primary care. *Lancet Oncol* 2020;21(6):748-50. doi: 10.1016/S1470-2045(20)30242-4 [published Online First: 2020/05/04]
11. Richards M, Anderson M, Carter P, et al. The impact of the COVID-19 pandemic on cancer care. *Nat Cancer* 2020:1-3. doi: 10.1038/s43018-020-0074-y [published Online First: 2020/08/25]
12. Neal RD, Tharmanathan P, France B, et al. Is increased time to diagnosis and treatment in symptomatic cancer associated with poorer outcomes? Systematic review. *Br J Cancer* 2015;112 Suppl 1:S92-107. doi: 10.1038/bjc.2015.48 [published Online First: 2015/03/04]
13. Maringe C, Spicer J, Morris M, et al. The impact of the COVID-19 pandemic on cancer deaths due to delays in diagnosis in England, UK: a national, population-based, modelling study. *Lancet Oncol* 2020;21(8):1023-34. doi: 10.1016/S1470-2045(20)30388-0 [published Online First: 2020/07/24]
14. Price S, Spencer A, Zhang X, et al. Trends in time to cancer diagnosis around the period of changing national guidance on referral of symptomatic patients: A serial cross-sectional study using UK electronic healthcare records from 2006-17. *Cancer Epidemiol* 2020;69:101805. doi: 10.1016/j.canep.2020.101805 [published Online First: 2020/09/13]
15. Watson J, Nicholson BD, Hamilton W, et al. Identifying clinical features in primary care electronic health record studies: methods for codelist development. *BMJ Open* 2017;7(11):e019637. doi: 10.1136/bmjopen-2017-019637 [published Online First: 2017/11/25]
16. National Institute for Health and Care Excellence. Suspected cancer: recognition and referral. NICE guideline [NG12], 2020.
17. Clinical Practice Research Datalink. CPRD; UK data driving real-world evidence [Available from: www.cprd.com2020.

For peer review only - http://bmjopen.bmj.com/site/about/guidelines.xhtml

UOB Confidential & Sensitive

18. NHS Digital. Quality and Outcomes Framework (QOF) business rules v42 2019-2020 baseline release, 2019/2020.
19. NHS Digital. Patients Registered at a GP Practice 2019-2020 [Available from: https://digital.nhs.uk/data-and-information/publications/statistical/patients-registered-at-a-gp-practice2020.
20. Price SJ, Stapley SA, Shephard E, et al. Is omission of free text records a possible source of data loss and bias in Clinical Practice Research Datalink studies? A case-control study. *BMJ Open* 2016;6(5):e011664. doi: 10.1136/bmjopen-2016-011664 [published Online First: 2016/05/15]
21. Williams R, Jenkins DA, Ashcroft DM, et al. Diagnosis of physical and mental health conditions in primary care during the COVID-19 pandemic: a retrospective cohort study. *Lancet Public Health* 2020;5(10):e543-e50. doi: 10.1016/S2468-2667(20)30201-2 [published Online First: 2020/09/27]
22. Hammersley V, Donaghy E, Parker R, et al. Comparing the content and quality of video, telephone, and face-to-face consultations: a non-randomised, quasi-experimental, exploratory study in UK primary care. *Br J Gen Pract* 2019;69(686):e595-e604. doi: 10.3399/bjgp19X704573 [published Online First: 2019/07/03]
23. Royal College of General Practitioners. Research and Surveillance Centre workload observatory 2020 [Available from: https://clininf.eu/index.php/rcgprscworkloadobservatory/.
24. Faber MJ, Burgers JS, Westert GP. A sustainable primary care system: lessons from the Netherlands. *J Ambul Care Manage* 2012;35(3):174-81. doi: 10.1097/JAC.0b013e31823e83a4 [published Online First: 2012/06/07]

For peer review only

For peer review only - http://bmjopen.bmj.com/site/about/guidelines.xhtml

UOB Confidential & Sensitive

Table 1: Characteristics of registered and consulting patients in participating practices

	All patients registered in July 2020 (N=126,466)		Patients consulting in April-July 2019 (N=69,254)		Patients consulting in April-July 2020 (N=56,314)	
	n	%	n	%	n	%
Age						
50-59 years	44,614	35.3%	20,830	30.1%	16,711	29.7%
60-69 years	33,338	26.4%	17,765	25.7%	14,252	25.3%
70-79 years	27,536	21.8%	17,512	25.3%	14,066	25.0%
80+ years	20,978	16.6%	13,147	19.0%	11,285	20.0%
Gender						
Male	61,798	48.9%	30,949	44.7%	25,129	44.6%
Female	64,668	51.1%	38,305	55.3%	31,185	55.4%
Index of multiple deprivation quintile						
1 (most deprived)	19,392	15.4%	10,877	15.8%	9,355	16.7%
2	15,907	12.7%	8,792	12.8%	7,403	13.2%
3	18,665	14.9%	10,197	14.8%	8,151	14.5%
4	28,169	22.4%	15,174	22.0%	12,236	21.8%
5 (least deprived)	43,500	34.6%	23,861	34.6%	18,888	33.7%
Missing	*833*		*353*		*281*	
Ethnicity*						
White	91,401	93.2%	52,262	93.3%	42,512	93.3%
Asian	2,507	2.6%	1,443	2.6%	1,161	2.5%
Black	2,963	3.0%	1,655	3.0%	1,374	3.0%
Mixed	1,000	1.0%	532	0.9%	425	0.9%
Other	227	0.2%	111	0.2%	89	0.2%
Missing	*28,368*		*13,251*		*10,753*	
Mental health status						
No MH conditions	111,143	87.9%	57,923	83.6%	45,885	81.5%
1+ MH condition	15,323	12.1%	11,331	16.4%	10,429	18.5%
Shielding status						
Not advised to shield	116,518	92.1%	61,417	88.7%	48,305	85.8%
Advised to shield	9,948	7.9%	7,837	11.3%	8,009	14.2%

*White includes British, white British, mixed British, and other white background. Black includes African, Caribbean, Somali and black British. Asian includes Indian, British Indian, Pakistani, Chinese, and other Asian background. Mixed includes white and black Caribbean, white and Asian, white and African, other mixed white, and other mixed background. Other includes, Turkish, Arab, Iranian, other ethnic non-mixed, and any other group. See supplemental file 1 for further details.

For peer review only - http://bmjopen.bmj.com/site/about/guidelines.xhtml

UOB Confidential & Sensitive

Table 2: Patients with any potential cancer indicator reported in April-July 2020 compared to 2019

	April-July 2019 (n=123,947)		April-July 2020 (n=126,466)		Change in proportion of patients with potential cancer indicators (2020 vs. 2019)		
	n	% [a]	n	% [a]	IRR	95% CI	p-value
Overall	21,252	17%	13,829	11%	0.64	0.62 to 0.67	<0.001
By Age category							0.136 [b]
50-59 years	6,217	14%	4,083	9%	0.65	0.61 to 0.69	<0.001
60-69 years	5,398	17%	3,368	10%	0.61	0.58 to 0.65	<0.001
70-79 years	5,457	20%	3,449	13%	0.62	0.59 to 0.67	<0.001
80+ years	4,180	20%	2,929	14%	0.68	0.64 to 0.73	<0.001
By Sex							0.467 [b]
Male	9,202	15%	5,905	10%	0.63	0.60 to 0.67	<0.001
Female	12,050	19%	7,924	12%	0.65	0.62 to 0.68	<0.001
By Ethnicity [c]							0.471 [b]
White	15,910	18%	10,340	11%	0.64	0.61 to 0.68	<0.001
Asian	590	24%	357	14%	0.58	0.50 to 0.69	<0.001
Black	710	24%	497	17%	0.68	0.58 to 0.80	<0.001
Mixed	193	20%	106	11%	0.55	0.42 to 0.70	<0.001
Other	40	18%	25	11%	0.60	0.36 to 1.00	0.050
By index of multiple deprivation quintile							0.585 [b]
1 (most deprived)	3,916	21%	2,662	14%	0.67	0.63 to 0.72	<0.001
2	2,978	19%	1,981	12%	0.64	0.60 to 0.69	<0.001
3	3,158	17%	2,052	11%	0.63	0.59 to 0.68	<0.001
4	4,607	17%	2,999	11%	0.63	0.59 to 0.67	<0.001
5 (least deprived)	6,469	15%	4,053	9%	0.62	0.58 to 0.67	<0.001
By Mental health status							<0.001 [b]
No MH conditions	17,338	16%	10,924	10%	0.62	0.60 to 0.64	<0.001
1+ MH condition	3,914	26%	2,905	19%	0.73	0.69 to 0.77	<0.001
By Shielding status							0.099 [b]
Not advised to shield	17,956	16%	11,564	10%	0.63	0.61 to 0.66	<0.001
Advised to shield	3,296	34%	2,265	23%	0.68	0.63 to 0.72	<0.001

IRR=incidence rate ratio. CI=Confidence interval. IRRs and corresponding 95% CIs and p-values from negative binomial models.

[a] Percentages are calculated out of all patients in each category.

[b] P-values presented on the blue rows are the p-values for the interaction between year and the given characteristic; incidence rate ratios only presented by each level of a characteristic if this p<0.05.

[c] White includes British, white British, mixed British, and other white background. Black includes African, Caribbean, Somali and black British. Asian includes Indian, British Indian, Pakistani, Chinese, and other Asian background. Mixed includes white and black Caribbean, white and Asian, white and African, other mixed white, and other mixed background. Other includes, Turkish, Arab, Iranian, other ethnic non-mixed, and any other group. See supplemental file 1 for further details.

For peer review only - http://bmjopen.bmj.com/site/about/guidelines.xhtml

UOB Confidential & Sensitive

Table 3: Consultations (per 1000 patients) with potential cancer indicators reported in April-July 2020 compared to 2019

	April-July 2019		April-July 2020		Change in rates of consultations with potential cancer indicators (2020 vs. 2019)		
GP CONSULTATIONS	n	%[a]	n	%[a]	IRR	95% CI	p-value
Overall	236.5	19%	176.4	15%	0.79	0.74 to 0.84	<0.001
By consultation provision							<0.001 [b]
Face-to-face	187.8	21%	25.4	17%	0.84	0.76 to 0.92	<0.001
Remote	48.7	13%	151.0	14%	1.17	1.07 to 1.29	0.001
NURSE/PARAMEDIC CONSULTATIONS	n	%	n	%	IRR	95% CI	p-value
Overall	53.6	9%	31.9	7%	0.69	0.56 to 0.85	<0.001
By consultation provision							<0.001 [b]
Face-to-face	50.2	10%	15.7	6%	0.61	0.44 to 0.83	0.002
Remote	3.4	7%	16.1	9%	1.60	1.10 to 2.33	0.014

IRR=incidence rate ratio. CI=Confidence interval. IRRs and corresponding 95% CIs and p-values from negative binomial models.

[a] Percentages are calculated out of all consultations in each category.

[b] P-values presented on the blue rows are the p-values for the interaction between year and consultation provision.

For peer review only - http://bmjopen.bmj.com/site/about/guidelines.xhtml

UOB Confidential & Sensitive

Table 4: Patients with individual potential cancer indicator reported in April-July 2020 compared to 2019

	April-July 2019 (n=123,947)		April-July 2020 (n=126,466)		
	n	%	n	%	IRR
Any potential cancer indicator	21,252	17.15%	13,829	10.93%	0.64
Most commonly reported indicators (≥0.5% of patients in 2019)					
Any commonly reported indicator	*16,632*	*13.42%*	*10,054*	*7.95%*	*0.59*
Chest infection	1,427	1.15%	370	0.29%	0.25
Fever	1,216	0.98%	443	0.35%	0.36
Cough	3,125	2.52%	1,397	1.10%	0.44
Fatigue	1,284	1.04%	655	0.52%	0.50
Urinary tract infection	760	0.61%	394	0.31%	0.51
Pain in shoulder	1,307	1.05%	785	0.62%	0.59
Proteinuria	697	0.56%	437	0.35%	0.61
Non-visible haematuria	979	0.79%	616	0.49%	0.62
Pain in abdomen	1,908	1.54%	1,301	1.03%	0.67
Haematuria	897	0.72%	612	0.48%	0.67
Shortness of breath	2,382	1.92%	1,650	1.30%	0.68
Pain in chest	1,180	0.95%	830	0.66%	0.69
Back pain/backache	2,659	2.15%	1,887	1.49%	0.70
Lower urinary tract symptoms	1,402	1.13%	1,027	0.81%	0.72
Compressed trachea	864	0.70%	638	0.50%	0.72
Diarrhoea	860	0.69%	645	0.51%	0.74
Less commonly reported indicators (0.1%-<0.5% of patients in 2019)					
Any less commonly reported indicator	*6,585*	*5.31%*	*5,008*	*3.96%*	*0.75*
Erectile dysfunction	363	0.29%	148	0.12%	0.40
Irritable Bowel Syndrome	194	0.16%	97	0.08%	0.49
Gynae abnormalities (e.g. menstrual disorders and unusual bleeding)	190	0.15%	99	0.08%	0.51
Pain in throat (e.g. tonsilitis)	201	0.16%	109	0.09%	0.53
Iron deficiency anaemias	477	0.38%	273	0.22%	0.56
Change in bowel habit	281	0.23%	161	0.13%	0.56
Pain in breast	170	0.14%	99	0.08%	0.57
Bruising	208	0.17%	126	0.10%	0.59
Prostate specific antigen raised/abnormal	173	0.14%	121	0.10%	0.69
Postmenopausal bleeding	144	0.12%	101	0.08%	0.69
Pain in pelvis	388	0.31%	280	0.22%	0.71
Pruritus	356	0.29%	259	0.20%	0.71
Vomiting	245	0.20%	180	0.14%	0.72
Dyspepsia	590	0.48%	450	0.36%	0.75
Distension of abdomen	142	0.11%	111	0.09%	0.77
Reflux	560	0.45%	439	0.35%	0.77
Lump in breast	208	0.17%	170	0.13%	0.80
Recurring infections	366	0.30%	304	0.24%	0.81
Rectal bleeding	357	0.29%	303	0.24%	0.83
Dysuria	196	0.16%	169	0.13%	0.85
Vaginal discharge	153	0.12%	134	0.11%	0.86
Dysphagia	171	0.14%	151	0.12%	0.87
Lump (excluding breast)	402	0.32%	375	0.30%	0.91

For peer review only - http://bmjopen.bmj.com/site/about/guidelines.xhtml

UOB Confidential & Sensitive

Nausea	147	0.12%	150	0.12%	1.00
Weight loss	218	0.18%	239	0.19%	1.07
Constipation	545	0.44%	655	0.52%	1.18
Rarely reported indicators (0.02- <0.1% of patients in 2019)					
Any rarely reported indicator	*909*	*0.73%*	*702*	*0.56%*	*0.76*
Limp/ gait abnormalities	25	0.02%	10	0.01%	0.39
Night sweats	75	0.06%	33	0.03%	0.43
Lymphadenopathy	86	0.07%	38	0.03%	0.43
Haemoptysis	62	0.05%	34	0.03%	0.54
Abnormal chest x-ray	28	0.02%	17	0.01%	0.60
Hoarseness of voice	101	0.08%	65	0.05%	0.63
Pain in testis	71	0.06%	46	0.04%	0.63
Pain in bones	118	0.10%	86	0.07%	0.71
Possible bone and soft tissue sarcoma symptoms (e.g. abnormal x-ray)	30	0.02%	22	0.02%	0.72
Chest/breathing abnormalities	26	0.02%	20	0.02%	0.75
Mass in neck	115	0.09%	89	0.07%	0.76
Mass in testis	102	0.08%	79	0.06%	0.76
Superior vena cava syndrome	39	0.03%	38	0.03%	0.95
Testis swelling/abnormalities	31	0.03%	32	0.03%	1.01
Appetite loss	49	0.04%	76	0.06%	1.52
Very rarely reported indicators (<0.02% of patients in 2019)					
*Any very rarely reported indicator**	*141*	*0.11%*	*108*	*0.09%*	*0.75*

IRR=Incidence rate ratio.

Note. Indicators are ordered from largest to smallest reduction (by IRR) within each commonality grouping. These figures only include indicators coded in routinely collected records using SNOMED codes. In some cases, GPs/nurses may enter symptoms in free text, and/or only code their diagnosis, which may lead to under-reporting here; this under-recording is likely to be similar in both years.

* Very rare indicators affecting <0.02% of patients (25 individuals) in April-July 2019 clubbing of fingers, ulceration of vulva, prostate abnormalities on digital rectal exam, nipple retraction, skin lesion, mass in thyroid, jaundice, spinal cord compression, mass in pelvis, faecal occult blood test, abnormal skin on breast, nipple discharge, haematemesis and 12 additional indicators with incidence <5 patients in April-July 2019.

For peer review only

Figure 1:
Title: Proportion of patients consulting and proportion consulting with potential cancer indicators
Legend:

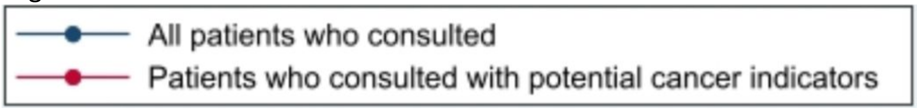

Figure 2:
Title: Number of consultations per '000 patients and proportion of those with potential cancer indicators
Legend:

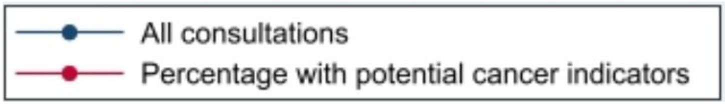

For peer review only - http://bmjopen.bmj.com/site/about/guidelines.xhtml

UOB Confidential & Sensitive

For peer review only

For peer review only - http://bmjopen.bmj.com/site/about/guidelines.xhtml
UOB Confidential & Sensitive

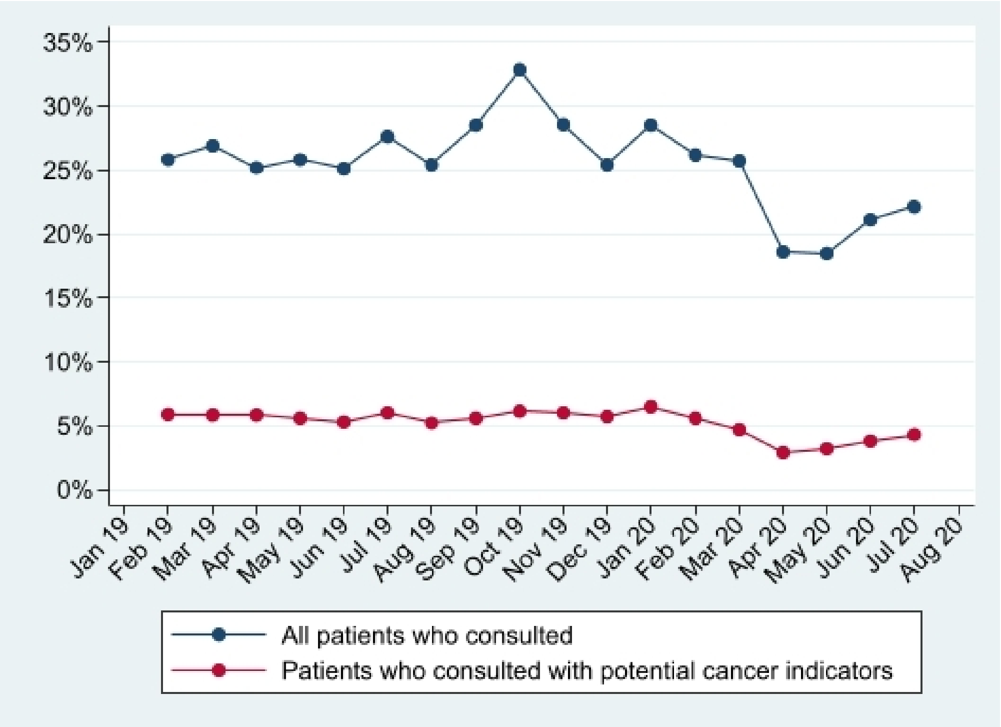

Figure 1: Proportion of patients consulting and proportion consulting with potential cancer indicators

139x101mm (96 x 96 DPI)

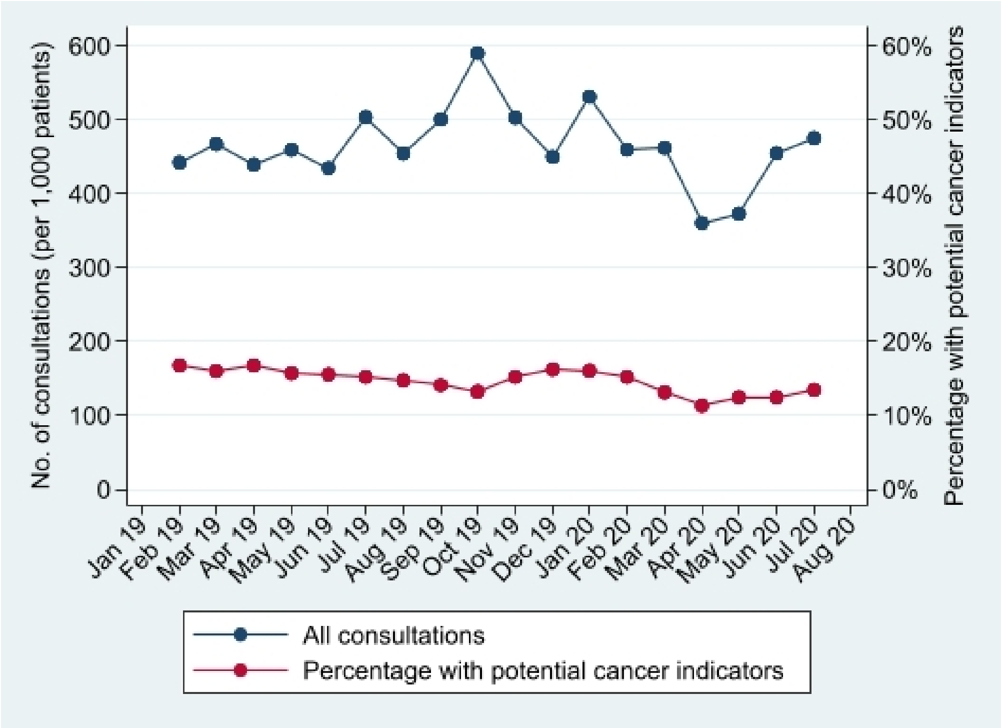

Figure 2: Number of consultations per '000 patients and proportion of those with potential cancer indicators

139x101mm (96 x 96 DPI)

Supplementary File 1: Data Rules

1. Data extraction and analysis

- ⇨ Data on patient characteristics, consultations and clinical codes for 21 practices were extracted by One Care.
- ⇨ We only extracted consultations added by clinicians. Administrator added consultations (e.g. e-consultations and documents uploaded) were not extracted.
- ⇨ Patient characteristics were extracted for all patients registered in July 2020.
- ⇨ Consultations were extracted for all patients registered in July 2020.
- ⇨ To calculate consultations per 1000 patients, we used the July 2020 patient list extracted by One Care as the denominator for the April-July 2020 consultations. For the April-July 2019 data, we adjusted this list size based on practice list size data (total list size of patients aged 50+ years for all 21 included practices) published by NHS digital. We calculated the quotient of July 2019/July 2020 list size, and used this as a multiplier for the denominator. We used this same method to individually calculate the offset for each practice for the negative binomial models. For the figures presenting data by month, we again used this same method but using monthly list sizes (for patients aged 50+ years) on NHS digital to calculate the multiplier for the denominator.

2. Consultation Type

We applied the following rules to identify consultation type:

Face-to-face: If

consultation category= "gp surgery" OR "face to face consultation" OR "face to face consultation with relative/carer" OR "emergency appointment" OR "extended hours consultation" OR "same day appointment" OR "urgent consultation".

Home: If
consultation category= "children's home visit note" OR "home visit note" OR "night visit note" OR "nursing home visit note" OR "residential home visit note" OR "twilight visit note"
OR
Clinical code text contains the words “home visit”.

Telephone: If
consultation category contains the words “telephone call” OR "telephone consultation” OR “enterprise consultation”[1]
OR
appointment type contains the words “telephone” OR “phonecall” OR “phone call” OR “telcon”
OR
appointment slot status= “telephone – complete”
OR

---

[1] These are consultations carried out in a GP surgery which is not the patients own surgery. We identified these as telephone consultation as we do not know anything more about the medium of these consultations and telephone was most likely. If clinical codes for video consultation were identified, this was coded to video instead, in accordance with the data rules shown in the following page.

For peer review only

Clinical code text contains the word “telephone” OR “telephone and/or video” OR “telephone/video” OR “telephone/ video” OR “telephone / video”

Video: If
consultation category= “consultation via video conference"
OR
Clinical code text= assessed by video consult" OR "consultation via video conference" OR "video consult"
E-consultation[2]: If
consultation category= “online communication” OR “consultation via multimedia” OR contains the word “e-mail”
OR
appointment type contains the words “e consult” OR “econsult” OR “e:consult”
OR
Clinical codes text contains the words “econsult” OR “e-consult” OR “e:consult” OR “e consult” OR “consultation via multimedia” OR “alert received from telehealth monitoring system”

We used the following rank order for determining consultation type: **Video, Telephone, E-consult, Home, Face-to-face** (E.g. if a patient has consultation type as “GP surgery” but has a clinical code for "video consult" then this is coded as video.)

Consultations were excluded as follows:

- ⇨ Non consultation activity (e.g. where the consultation category was “administration note”, “inbound document”, and “scanned document”).
- ⇨ Records which were appearing as consultations but were actually SMS messages. Identified as: *Consultation type*= ”Face-to-face” AND *consultation category*=”gp surgery” AND the consultation has a maximum of 3 clinical codes associated to it, and one is “patient mobile telephone number” or “patient telephone number”, and one is “sms (short message service) text message sent to patient” or “sms text message sent to patient” or “sms text sent to patient”
- ⇨ Consultations which the patient did not attend, or did not answer the phone: Identified by (*Clinical code text* contains the words “did not attend” OR “failed encounter” OR “dna” AND *clinical code text* does not equal “dna studies” OR “dnacpr”) OR (*appointment slot status* = “telephone - not in” OR “dna” OR “walked out”).

3. Staff type

Codes were mapped for staff type as follows:

GP: Assistant GP, Associate practitioner - general practitioner, General medical practitioner, GP registrar, Locum GP, Salaried general practitioner, Sessional GP

[2] Because we only included consultations added by clinicians, not administrators, these only include e-consultations which clinicians added to the patient record. E-consultations added by administrators which covert to a telephone call (added by a GP) or dealt with by a task are not included.

For peer review only

Nurse/Paramedic: Advanced practitioner, Associate practitioner – nurse, Community nurse, Community practitioner, Enrolled nurse, Modern matron, Nurse consultant, Nurse manager, Paramedic, Paramedic specialist practitioner, Sister/charge nurse, Specialist practitioner, Staff nurse.

An Allied Professional Staff Type was also created. However, as the numbers of consultations were very small, this was not used in our analysis.

4. Ethnicity

Codes were mapped for ethnicity as follows:

- **White**: 44 different codes. Over 90% of patients were covered by the codes "White British", “British or mixed British” or "other white background."
- **Black/African/Caribbean/Black British**: 36 different codes. Over 90% were coded as “African”, “Caribbean”, “black African", “black Caribbean”, "Somali”, or “black British”.
- **Asian/Asian British**: 49 different codes. 90% of patients were covered by the codes “Indian or British Indian”, “other Asian background”, “Pakistani or British Pakistani", “Chinese”, “Bangladeshi or British Bangladeshi”, “Indian” or “Pakistani”.
- **Mixed/Multiple ethnic groups**: 27 different codes: 90% covered by “white and black Caribbean”, "other mixed background”, “white and Asian”, "white and black African”, “other mixed white”, “black and white”.
- **Other**: 40 codes including: “any other group”, “Turkish”, “Arab”, “other ethnic non-mixed (nmo)”, “Iranian”, "gypsy/Romany”, “Kurdish”, “Latin American”, “Albanian", “other European (nmo)”.

For peer review only

STROBE Statement—Checklist of items that should be included in reports of ***cohort studies***

	Item No	Recommendation	Page
**Title and abstract**	1	(*a*) Indicate the study's design with a commonly used term in the title or the abstract	2
		(*b*) Provide in the abstract an informative and balanced summary of what was done and what was found	2
		**Introduction**	
Background/rationale	2	Explain the scientific background and rationale for the investigation being reported	4
Objectives	3	State specific objectives, including any prespecified hypotheses	4
		**Methods**	
Study design	4	Present key elements of study design early in the paper	4-6
Setting	5	Describe the setting, locations, and relevant dates, including periods of recruitment, exposure, follow-up, and data collection	4
Participants	6	(*a*) Give the eligibility criteria, and the sources and methods of selection of participants. Describe methods of follow-up	4
		(*b*) For matched studies, give matching criteria and number of exposed and unexposed	-
Variables	7	Clearly define all outcomes, exposures, predictors, potential confounders, and effect modifiers. Give diagnostic criteria, if applicable	4-6
Data sources/ measurement	8*	For each variable of interest, give sources of data and details of methods of assessment (measurement). Describe comparability of assessment methods if there is more than one group	4-6
Bias	9	Describe any efforts to address potential sources of bias	5
Study size	10	Explain how the study size was arrived at	-
Quantitative variables	11	Explain how quantitative variables were handled in the analyses. If applicable, describe which groupings were chosen and why	5-6
Statistical methods	12	(*a*) Describe all statistical methods, including those used to control for confounding	5-6
		(*b*) Describe any methods used to examine subgroups and interactions	5-6
		(*c*) Explain how missing data were addressed	-
		(*d*) If applicable, explain how loss to follow-up was addressed	-
		(*e*) Describe any sensitivity analyses	-
		**Results**	
Participants	13*	(a) Report numbers of individuals at each stage of study—eg numbers potentially eligible, examined for eligibility, confirmed eligible, included in the study, completing follow-up, and analysed	6-7
		(b) Give reasons for non-participation at each stage	-
		(c) Consider use of a flow diagram	-
Descriptive data	14*	(a) Give characteristics of study participants (eg demographic, clinical, social) and information on exposures and potential confounders	6, Table 1
		(b) Indicate number of participants with missing data for each variable of interest	Table 1
		(c) Summarise follow-up time (eg, average and total amount)	6-7
Outcome data	15*	Report numbers of outcome events or summary measures over time	6-7

For peer review only - http://bmjopen.bmj.com/site/about/guidelines.xhtml

Main results	16	(*a*) Give unadjusted estimates and, if applicable, confounder-adjusted estimates and their precision (eg, 95% confidence interval). Make clear which confounders were adjusted for and why they were included	6-7
		(*b*) Report category boundaries when continuous variables were categorized	6-7 Tables 1,2,3
		(*c*) If relevant, consider translating estimates of relative risk into absolute risk for a meaningful time period	-
Other analyses	17	Report other analyses done—eg analyses of subgroups and interactions, and sensitivity analyses	6-7
**Discussion**			
Key results	18	Summarise key results with reference to study objectives	7
Limitations	19	Discuss limitations of the study, taking into account sources of potential bias or imprecision. Discuss both direction and magnitude of any potential bias	8
Interpretation	20	Give a cautious overall interpretation of results considering objectives, limitations, multiplicity of analyses, results from similar studies, and other relevant evidence	7-9
Generalisability	21	Discuss the generalisability (external validity) of the study results	8
**Other information**			
Funding	22	Give the source of funding and the role of the funders for the present study and, if applicable, for the original study on which the present article is based	10

*Give information separately for exposed and unexposed groups.

Note: An Explanation and Elaboration article discusses each checklist item and gives methodological background and published examples of transparent reporting. The STROBE checklist is best used in conjunction with this article (freely available on the Web sites of PLoS Medicine at http://www.plosmedicine.org/, Annals of Internal Medicine at http://www.annals.org/, and Epidemiology at http://www.epidem.com/). Information on the STROBE Initiative is available at http://www.strobe-statement.org.

For peer review only - http://bmjopen.bmj.com/site/about/guidelines.xhtml

BMJ Open

Changes in presentations with features potentially indicating cancer in primary care during the COVID-19 pandemic: a retrospective cohort study

Journal:	*BMJ Open*
Manuscript ID	bmjopen-2021-050131.R1
Article Type:	Original research
Date Submitted by the Author:	17-Apr-2021
Complete List of Authors:	Scott, Lauren; University Hospitals Bristol and Weston NHS Foundation Trust, National Institute for Health Research Applied Research Collaboration West; University of Bristol Medical School, Population Health Sciences<br>Murphy, Mairead; University of Bristol Faculty of Medicine and Dentistry, Centre for Academic Primary Care<br>Price, Sarah; University of Exeter Medical School,<br>Lewis, Rhys; One Care<br>Denholm, Rachel; University of Bristol, Centre for Academic Primary Care<br>Horwood, Jeremy; University of Bristol, UK, School of Social and Community Medicine<br>Palmer, Tom; University of Bristol Medical School, Population Health Sciences<br>Salisbury, Chris; University of Bristol, Centre for Academic Primary Care
**Primary Subject Heading**:	General practice / Family practice
Secondary Subject Heading:	General practice / Family practice, Oncology
Keywords:	PRIMARY CARE, COVID-19, ONCOLOGY

SCHOLARONE™
Manuscripts

I, the Submitting Author has the right to grant and does grant on behalf of all authors of the Work (as defined in the below author licence), an exclusive licence and/or a non-exclusive licence for contributions from authors who are: i) UK Crown employees; ii) where BMJ has agreed a CC-BY licence shall apply, and/or iii) in accordance with the terms applicable for US Federal Government officers or employees acting as part of their official duties; on a worldwide, perpetual, irrevocable, royalty-free basis to BMJ Publishing Group Ltd ("BMJ") its licensees and where the relevant Journal is co-owned by BMJ to the co-owners of the Journal, to publish the Work in this journal and any other BMJ products and to exploit all rights, as set out in our licence.

The Submitting Author accepts and understands that any supply made under these terms is made by BMJ to the Submitting Author unless you are acting as an employee on behalf of your employer or a postgraduate student of an affiliated institution which is paying any applicable article publishing charge ("APC") for Open Access articles. Where the Submitting Author wishes to make the Work available on an Open Access basis (and intends to pay the relevant APC), the terms of reuse of such Open Access shall be governed by a Creative Commons licence – details of these licences and which Creative Commons licence will apply to this Work are set out in our licence referred to above.

Other than as permitted in any relevant BMJ Author's Self Archiving Policies, I confirm this Work has not been accepted for publication elsewhere, is not being considered for publication elsewhere and does not duplicate material already published. I confirm all authors consent to publication of this Work and authorise the granting of this licence.

For peer review only

Changes in presentations with features potentially indicating cancer in primary care during the COVID-19 pandemic: a retrospective cohort study

Lauren J Scott[1,2], Mairead Murphy[3], Sarah Price[4], Rhys Lewis[5], Rachel Denholm[3,6], Jeremy Horwood[1,3], Tom Palmer[2,7], Chris Salisbury[1,3]

[1] National Institute for Health Research Applied Research Collaboration West (NIHR ARC West), University Hospitals Bristol and Weston NHS Foundation Trust, Bristol, UK

[2] Population health Sciences, Bristol Medical School, University of Bristol, Bristol, UK

[3] Centre for Academic Primary Care (CAPC), Bristol Medical School, University of Bristol, Bristol, UK

[4] Exeter Medical School, University of Exeter, Exeter, UK

[5] One Care, Bristol, UK

[6] National Institute for Health Research Biomedical Research Centre, University of Bristol, Bristol, UK

[7] MRC Integrative Epidemiology Unit, University of Bristol, Bristol, UK

Name	Qualification	Position	Institution	Email
Lauren J Scott	MSc	Senior Research Associate	University of Bristol	Lauren.Scott@bristol.ac.uk
Mairead Murphy	PhD	Research Fellow	University of Bristol	Mairead.murphy@bristol.ac.uk
Sarah Price	PhD	Research Fellow	University of Exeter	S.J.Price@exeter.ac.uk
Rhys Lewis	MPH	Senior Business Analyst	One Care	Rhys.lewis@onecare.org.uk
Rachel Denholm	PhD	Lecturer	University of Bristol	R.denholm@bristol.ac.uk
Jeremy Horwood	PhD	Associate Professor	University of Bristol	J.horwood@bristol.ac.uk
Tom Palmer	PhD	Senior Lecturer	University of Bristol	tom.palmer@bristol.ac.uk
Chris Salisbury	MD, FRCGP	Professor	University of Bristol	C.salisbury@bristol.ac.uk

Corresponding author:
mairead.murphy@bristol.ac.uk
Centre for Academic Primary Care
Bristol Medical School
Bristol BS8 2 PS

Word count=4,026.

For peer review only - http://bmjopen.bmj.com/site/about/guidelines.xhtml

UOB Confidential & Sensitive

For peer review only

Abstract

Objectives
To investigate how the COVID-19 pandemic affected the number of people aged 50+ presenting to primary care with features which could potentially indicate cancer, and to explore how reporting differed by patient characteristics and in face-to-face vs. remote consultations.

Design, Setting and Participants
A retrospective cohort study of GP, nurse, and paramedic primary-care consultations in 21 practices in South-West England covering 123,947 patients. The models compared potential cancer indicators reported in April – July 2019 with April – July 2020.

Main outcome measures
Potential indicators of cancer were identified using code lists for symptoms, signs, test results and diagnoses listed in the National Institute for Health and Care Excellence (NICE) suspected-cancer referral guidance (NG12).

Results
During April-July 2019, 17% of registered patients aged 50+ years reported a potential cancer indicator in a consultation with a GP or nurse. During April-July 2020 this reduced to 11% (IRR 0.64, 95% CI 0.62 to 0.67, p<0.001). Reductions in potential cancer indicators were stable across age group, sex, ethnicity, index of multiple deprivation (IMD) quintile, and shielding status, but less marked in patients with mental health conditions than without (IRR=0.75, 95% CI 0.72 to 0.79, interaction p<0.001). Proportions of GP consultations with potential indicators of cancer reduced between 2019 and 2020 for face-to-face consultations (IRR=0.84, 95% CI 0.76 to 0.92, p<0.001) and increased for remote consultations (IRR=1.17, 95% CI 1.07 to 1.29, p=0.001), though it remained lower in remote consulting than face-to-face in April-July 2020. This difference was greater for nurse/paramedic consultations (face-to-face: IRR=0.61, 95% CI 0.44 to 0.83, p=0.002; remote: IRR=1.60, 95% CI 1.10 to 2.333, p=0.014).

Conclusion
The number of patients consulting with presentations that could potentially indicate cancer reduced during the first-wave of the COVID-19 pandemic. Patients should be encouraged to continue contacting primary care for persistent signs and symptoms, and GPs and nurses should be encouraged to probe patients for further information during remote consulting, in the absence of non-verbal cues.

Key words: COVID-19, General Practice, Cancer

Strengths and limitations of this study

- We were able to use a large number of patients (>126,000) aged 50+ years from a diverse range of backgrounds, and a comprehensive list of potential cancer indicators.
- We were able to identify the number of patients presenting to primary care with symptoms, signs, test results or diagnoses which could potentially indicate cancer, and to explore how this reporting differed by patient characteristics and consultation provision.
- We were unable to take account of whether symptoms were persistent or unexplained, which may inflate our estimates of potential cancer indicators.
- Some symptoms and signs may have been reported in free text rather than using clinical codes or may have been recorded in administration notes not associated with consultations.

For peer review only - http://bmjopen.bmj.com/site/about/guidelines.xhtml

UOB Confidential & Sensitive

Introduction

In March 2020, the World Health Organisation declared COVID-19 a pandemic[1], and the UK went into lockdown; the public were instructed to "Stay at home, protect the NHS, and save lives".[2] Primary care providers were advised to provide all consultations remotely, unless a face-to-face consultation was urgently required,[3] in order to reduce contact and the spread of the virus. Patients at particularly high risk of severe COVID-19 due to age or pre-existing health conditions were advised to 'shield' and avoid all but essential contact.

GP and nurse consulting rates markedly dropped in April-May 2020 during the period of UK lockdown, but had largely returned to normal by July 2020,[4] with the majority of consultations carried out remotely (mostly via telephone).

There are fears that reduced consulting during the lockdown period and changes in consultation provision may have adversely impacted on cancer detection.[5] There was a 63% reduction in skin cancer diagnosis in England in the first three month of the pandemic[6] and 3500 fewer colorectal cancer diagnoses than expected in the first six months.[7] This pattern is not limited to the UK. An analysis on the Danish registry showed cancer diagnosis in Denmark dropped by 33% from March to May 2020[8] and similar reductions have been observed in the Netherlands.[9]

These concerning reductions in diagnoses are likely to result from a combination of factors, including temporary suspensions in cancer screening services,[10 11] reduced capacity in secondary care and less reporting of potential cancer symptoms in primary care. A Cancer Research UK survey suggested GPs perceived they received fewer reports of cancer symptoms, particularly from older people, after the pandemic than before.[5] This could be related to changes in the willingness of patients to disclose symptoms, or changes in patient or doctor behaviour associated with the shift to remote consultations. Reduced symptom reporting could result in late diagnoses, increased workload for cancer services, and poorer patient outcomes.[12] A modelling study showed that delays in cancer diagnosis in this period could result in approximately 3,330 deaths for breast, colorectal, lung and oesophageal cancer alone.[13]

Aims: The aim of this study was to investigate how the COVID-19 pandemic affected the number of people aged 50 years and over presenting to primary care with symptoms, signs, test results or diagnoses which could potentially indicate cancer, and to explore how this reporting differed by patient characteristics and consultation provision (i.e. face-to-face vs. remote).

Methods

Design and setting

This was a retrospective cohort study of primary care consultations in 21 practices in Bristol, North Somerset, and South Gloucestershire, a region in the South West of England. Data were extracted for the RAPCI Study (Rapid COVID-19 intelligence to improve primary care response), a mixed-methods study on how general practices coped with rapid change to remote consulting in the initial months of the COVID-19 pandemic. The main results are reported elsewhere.[4]

Data

All 82 practices in Bristol, North Somerset, and South Gloucestershire were approached by the Clinical Commissioning Group (CCG) with information about the study. Practices returned expressions of interest to the researchers who selected 21 practices, aiming for a variation of deprivation, ethnic mix, practice size and locality. All participating practices then agreed for

For peer review only - http://bmjopen.bmj.com/site/about/guidelines.xhtml

UOB Confidential & Sensitive

anonymised data to be extracted directly from the patient record by One Care, the GP federation in Bristol, North Somerset and South Gloucestershire CCG. One Care provide technical support and management information services to GP practices in the CCG and have the ability to extract routine data from the patient record via the EMIS enterprise search and report module. All practices use the same EMIS electronic medical records system. Data on all patients registered on 1st July 2020 were included. Patient data included demographics (age, sex, ethnicity, and deprivation) and clinical characteristics (such as mental health and shielding status). Data was obtained on all consultations added to the system by clinical staff, and all clinical codes added during those consultations between February 2019 and July 2020 inclusive. We extracted data from February 2019 so that we had a full 18-months to examine trends for the main study. For the analyses in this paper, the period April-July 2020 (i.e. the period since the start of UK lockdown) was compared to the same period the previous year (April-July 2019). For this paper, we restricted the analysis to patients aged 50 years and over as cancer is relatively rare in younger age groups.

Consultations
A consultation was defined as an interaction between a patient and a GP, nurse, or paramedic working in general practice. We focussed on GP and nurse/paramedic consultations because the number of cancer indicators reported in consultations with other health professionals was very small. We excluded consultations recorded by administrators or other health care professionals, and administrative tasks recorded by clinicians. Remote consultations were defined as those carried out by telephone, video, or e-consultation. Face-to-face consultations were those carried out in GP practices as well as visits to patients' homes or nursing homes. See supplemental file 1 for further details.

Outcomes
Pre-existing code lists[14 15] were used to identify records of potential cancer indicators associated with a consultation. The indicators were collated from the clinical features of undiagnosed cancer (symptoms, signs, abnormal test results or diagnoses) listed in the National Institute for Health and Care Excellence guidance on the recognition and referral of suspected cancer (NG12)[16], using robust methods.[15] Lists were developed using Read codes and subsequently mapped to SNOMED CT codes using medical dictionaries provided by the Clinical Practice Research Datalink for its AURUM dataset.[17] There were a small number of Read codes in the pre-defined list which do not have equivalent SNOMED CT codes; the frequency with which these codes were used was so low as to be materially insignificant. New SNOMED codes added since the changeover from Read codes to SNOMED did not appear in our pre-defined lists so we checked this for common indicators cough and back pain, and found new codes were used very rarely.
SNOMED CT codes were used for this analysis. We excluded the indicator for new-onset diabetes (defined as a diabetes code in the absence of any diabetes monitoring in the previous 2 years), because we did not have the necessary patient records required to calculate this.

A binary variable for 'any potential cancer indicators' was derived based on whether a consultation contained at least one potential cancer indicator code (Yes) or did not (No). Using this consultation data, the number of patients reporting potential cancer indicators was calculated.

Each potential cancer indicator was categorised based on how many patients reported it in April-July 2019: most commonly (≥0.5%), less commonly (0.1% to <0.5%), rarely (0.02% to <0.1%), and very rarely reported (<0.02%). We felt it was important to separate these, as the most commonly reported indicators include symptoms which often indicate acute illness or chronic illnesses other than cancer (e.g. cough), whereas the less commonly/rarely reported indicators are more likely to be associated with cancer (e.g. weight loss, lumps and masses).

For peer review only - http://bmjopen.bmj.com/site/about/guidelines.xhtml

UOB Confidential & Sensitive

Explanatory variables
Age (in July 2020) was split into four categories: 50-59 years, 60-69 years, 70-79 years, and 80+ years old. Deprivation quintiles (measured by the index of multiple deprivation score; IMD) were calculated using the IMD deciles recorded in the patient record, which are based on Lower Super Output Areas of residence. Ethnicity was derived by mapping ethnicity descriptions in primary care records to one of five ethnicity categories: white, Asian, Black, mixed, and other (see supplemental file 1). Presence of a mental health condition was defined as either severe mental illness (defined according to the Quality and Outcomes Framework (QOF) rules[18]), diagnosed depression, or prescribed anti-depressants (excluding tricyclics, commonly used for non-mental health related conditions) in the three months prior to July 2020. We these conditions because they are part of QOF and are therefore likely to be coded more reliably within GP medical records than other conditions. Sex and shielding status (as of July 2020) were obtained directly from patients' primary care records.

Statistical analysis
The number and percentage of patients reporting potential cancer indicators during our reporting periods are presented. In addition, consultation rates (and percentages with potential cancer indicators) are reported per 1,000 registered patients. Practice list sizes were based on July 2020 data, and adjusted to account for historic list sizes using NHS digital data[19] ('adjusted list size'; see supplemental file 1).

Changes in proportions of patients presenting (per practice) with any potential cancer indicator in April-July 2020 compared to April-July 2019 were investigated using negative binomial regression models with counts of patients presenting with potential cancer indicators as the outcome; incidence rate ratios (IRRs) and 95% confidence intervals (CIs) are reported. Consultation year was fitted as a fixed effect, GP practice as a random effect, and adjusted practice list size (per level of covariate where appropriate) as the offset. Fixed effects for each of the categorical patient characteristics of interest (age, sex, ethnicity, IMD quintile, mental health status, and shielding status), along with an interaction term between each of these covariates and consultation year, were separately fitted to the model; interaction p-values are presented and changes in proportions of patients with potential cancer indicators are only presented separately for each level of a covariate if the interaction p-value was <0.05. Model validity was checked using standard methods; outliers which disrupted model fit were removed.

To investigate the effect of consultation provision (i.e. face-to-face vs. remote consulting), we looked at proportions of consultations with potential cancer indicators (out of all consultations) rather than proportions of patients. Separate models were fitted for consultations with GPs and nurses/paramedics. Models were similar to those described above, with number of consultations as the outcome, a fixed effect for consultation provision and an interaction with year, GP practice as a random effect, and total numbers of consultations per practice per level of consultation provision as the offset.

For individual potential cancer indicators, unadjusted IRRs for the percentage of patients consulting in April-July 2020 compared to 2019 are presented to help interpretation. 95% confidence intervals are included to indicate uncertainty but, owing to the large number of indicators and resultant issues with multiple testing, no multivariate modelling was performed. Indicators with p<0.0008 have been highlighted. This p-value threshold was calculated using the Bonferroni correction, which reduces the standard 0.05 by a factor of the number of tests performed to allow for multiple testing. Within the individual cancer indicators we highlighted eight alarm symptoms: visible haematuria, rectal

For peer review only - http://bmjopen.bmj.com/site/about/guidelines.xhtml
UOB Confidential & Sensitive

bleeding, haematemesis, haemoptysis, jaundice, breast lump, postmenopausal bleeding in women aged 55 and over, and dysphagia. These are symptoms that warrant investigation in their own right because of their predictive power for cancer. [16]

Stata 15.1 was used to conduct all data checking, cleaning, and analyses.

Patient and Public involvement (PPI)
A PPI group reviewed the RAPCI proposal before submission, agreed with the importance of the study and commented on the plain language summary. A meeting was also held to discuss preliminary findings of the main study[4], where a number of points were raised which supported the current work. These included participants highlighting that they felt things were missed during their telephone consultations because the GP could not see them.

Results

The 21 GP practices recruited covered 25% of Bristol, North Somerset and South Gloucestershire CCG. Selection of practices aimed to be representative of the region in terms of deprivation, size, location and ethnicity. The practices covered 350,966 registered patients (as of July 2020). Of these 123,947 were over 50.

During April-July 2019, of the 123,947 registered patients over 50, 69,254 (56%) consulted, and 21,252 (17%) of patients reported a potential cancer indicator. In April-July 2020, of 126,466 registered patients over 50, 56,314 (45%) consulted and 13,829 (11%) reported an indicator. This comprised 1837 consultations per 1000 patients registered (with indicators reported in 16% of consultations) in April-July 2019 and 1663 consultations per 1000 patients (with indicators reported in 13% of consultations) in April-July 2020. Figures 1 and 2 demonstrate that patients consulting, and consultation rates, substantially dropped in April 2020 (following UK lockdown), and while consultation rates had recovered by July 2020, the proportion of patients who consulted had not. Further, the percentage of patients reporting potential cancer indicators (Figure 1), and percentage of consultations with indicators reported (Figure 2), also dropped in April 2020. Both had increased by July 2020 (to 4.3% of patients and 13.5% of consultations), but neither had recovered to pre-COVID levels (6.0% of patients and 15.2% of consultations in July 2019). Patients who consulted in 2020 were of similar age, sex, IMD quintile and ethnicity as those consulting in 2019, but may have been more likely to have a mental health condition or be shielding (Table 1).

There was a 36% reduction in patients who reported potential cancer indicators in April-July 2020 compared to 2019 (IRR 0.64, 95% CI 0.62 to 0.67, p<0.001; Table 2). This reduction was stable across age group (interaction p-value=0.136), sex (p=0.467), ethnicity (p=0.471), IMD quintile (p=0.585), and shielding status (p=0.099), but differed by mental health status (p<0.001; Table 2). The reduction in reporting of these indicators was less pronounced in patients with a mental health condition (IRR 0.73, 95% CI 0.69 to 0.77, p<0.001) than patients without a mental health condition (IRR 0.63, 95% CI 0.61 to 0.66, p<0.001; Table 2).

The proportion of consultations where potential cancer indicators were reported differed by consultation provision for both GP and nurse/paramedic consultations (both p<0.001; Table 3). In GP consultations, proportions of face-to-face consultations with indicators reported dropped between 2019 and 2020 (IRR 0.84, 95% CI 0.76 to 0.92, p=<0.001) and increased for remote consultations (IRR 1.17, 95% CI 1.07 to 1.29, p=0.001; Table 3). For nurse/paramedic consultations, this finding was even more extreme (IRR 0.61, 95% CI 0.44 to 0.83, p=0.002 for face-to-face consultations; IRR 1.60,

For peer review only - http://bmjopen.bmj.com/site/about/guidelines.xhtml

UOB Confidential & Sensitive

95% CI 1.10 to 2.33, p=0.014 for remote consultations). Despite this increase in the proportion of potential cancer indicators in remote consultations, in both years GP remote consultations were less likely to report potential cancer indicators compared with GP face-to-face consultations (Table 3).

The proportion of patients reporting all commonly reported indicators reduced between the two periods (unadjusted IRR=0.59), with the biggest reductions in chest infections (IRR=0.25), fever (IRR=0.36), coughs (IRR=0.44) and fatigue (IRR=0.50; Table 4). For less commonly reported and rarely reported indicators, the reductions were less marked (IRR=0.75 and 0.76, respectively). Erectile dysfunction (IRR=0.40), irritable bowel syndrome (IRR=0.49), limp/gait abnormalities (IRR=0.39), night sweats (IRR=0.43) and lymphadenopathy (IRR=0.43) saw the largest reductions in these groups. Less common indicators which were similar or even increased were: nausea (IRR=1.00), weight loss (IRR=1.07) and constipation (IRR=1.18). All of the eight alarm symptoms reduced in frequency. Only haematuria (IRR=0.67) saw a significant reduction after correcting for multiple testing (i.e. p<0.0008), but at an aggregated level there was a significant reduction in patients who reported at least one cancer alarm symptom (IRR 0.73, 95% CI 0.68 to 0.79, p<0.0008; Table 4).

Discussion

Summary of findings

In April 2020, coinciding with UK lockdown, both the proportion of patients consulting, and those presenting with features potentially indicating cancer reduced compared to April 2019. By July 2020, although overall consultation rates had returned to previous levels, the proportion of patients consulting, and the proportion with potential cancer indicators remained lower than the previous year. The reduction in potential cancer indicator reporting did not differ by age, sex ethnicity, deprivation, or shielding status, but was less evident in patients with mental health conditions than those with no mental health conditions. Our results show that, despite an increase in consultations (for any reason) in patients aged 85+ and patients advised to shield (shown in our previous paper[4]), these have not resulted in higher levels of potential cancer indicator reporting in these groups, perhaps indicating these consultations were for routine assessment rather than to address new issues. Patients with mental health conditions did, however, have a smaller reduction in presentations with features potentially indicating cancer than other patients, perhaps as a result of this increased focus on them from GPs.[4] Between 2019 and 2020, there was a large reduction in face-to-face consultations which involved reporting of cancer indicators, which was not fully compensated for by an increase in reporting of these indicators in remote consultations. Of the most commonly reported indicators, chest infections, fever, coughs and fatigue reduced most dramatically. The reduction in "alarm" indicators (23%) was less pronounced than the overall reduction (36%) but was still significant (P<0.0008).

Strengths and limitations

To our knowledge, this is the first paper to assess the effect of the COVID-19 pandemic on potential cancer indicator reporting in UK primary care. The analysis is based on a comprehensive list of potential cancer indicators, developed and validated by Price, which has been used in previous research and is available on request.[14 15] Our analysis included a large number of patients (>126,000) aged 50+ years from a diverse range of backgrounds, and as changes in service provision due to the pandemic have affected the whole country, our findings are likely to be generalisable to other areas

For peer review only - http://bmjopen.bmj.com/site/about/guidelines.xhtml

UOB Confidential & Sensitive

of England. As all patients were included, there was no selection bias within practices. We endeavoured to select practices that were representative of the region but, as participation was voluntary, there may have been some selection bias at a practice level. However, this is unlikely to have changed the conclusions regarding reductions in potential cancer indicators, as there is no reason to suppose research active practices would have a greater reduction than other practices. The representativeness of the data is best demonstrated with reference to the full RAPCI dataset (350,966 patients) not just the sample of 50+ patients in this dataset. The age and gender profile of the RAPCI data are representative of the UK. The proportion of ethnic minorities in the RAPCI data (12%) is representative of the CCG, but slightly less than the UK as a whole.[20] The deprivation mix was slightly polarised; a fully representative sample would have approximately 20% in each quintile. The full RAPCI dataset had 20% in the bottom and fourth quintile but 27% in the most affluent so the second and third quintiles were slightly under-represented. Except for ethnicity, missing data about patient characteristics were low. 26% of ethnicity data was missing in the RAPCI dataset (22% in the over 50s subset used for this paper). This may have led to information bias.

There are several limitations which pertain to the recording of potential cancer indicators. Firstly, some symptoms and signs may have been reported in free text rather than using clinical codes,[21] or may have been recorded in administration notes not associated with consultations. Further, as described in the methods, there were a small number of Read codes in the pre-defined list which do not have equivalent SNOMED CT codes, although the frequency with which these codes were used was so low as to be materially insignificant. These issues may have resulted in systematic under-reporting of potential cancer indicators; however, this is likely to be similar in both years and therefore should not affect the comparative findings. A further limitation was that new SNOMED codes added since the changeover from Read codes to SNOMED did not appear in our pre-defined lists, an issue which would affect April-July 2020 but not 2019; however, in checking the common indicators cough and back pain, we found new codes were used very rarely (data not shown). Finally, for indicators to be of concern as possible harbingers of cancer, they are sometimes qualified by age at onset, or as persistent or unexplained in National Institute for Health and Care Excellence suspected-cancer guidance (NG12). Therefore, we limited our analysis to patients over 50 years of age (because cancer is more common in older patients) but were unable to take account of whether symptoms were persistent or unexplained. This limitation applies to other studies based on routine records and using similar methods[14], but the effect may be to inflate our estimates of potential cancer indicators.

Comparison with other literature

Few other studies have looked at the content of primary care consultations following UK lockdown during the COVID 19 pandemic. Analysis of primary care data from a deprived urban population found that diagnoses of common conditions decreased substantially between March and May 2020, suggesting a large number of patients may have undiagnosed conditions resulting from changes in access post-lockdown.[22] Our study adds to these findings for cancer-specific diagnoses.

Cancer screening programmes, estimated to account for about 5% of cancer diagnoses, were suspended in the wake of the pandemic.[10] This made symptom-based diagnosis all the more urgent. UK lockdown could have affected symptom-based reporting via the move to remote consulting. Research has shown that telephone and video consultations result in fewer problems being identified than face-to-face consultations.[23] Lockdown could also have influenced patients to only contact primary care if they thought their problem was serious, which is likely to be reflected in the reduced proportion of patients consulting found in our study.

For peer review only - http://bmjopen.bmj.com/site/about/guidelines.xhtml

UOB Confidential & Sensitive

Early in the pandemic, GPs predicted that patients with well-recognised red-flag symptoms, such as a new lump or rectal bleeding, would continue to present to primary care, but that vaguer cancer symptoms such as fatigue, change in bowel habit, and weight loss might be dismissed by the patient as trivial and not presented to primary care.[10] This was supported by our findings, which show that the most common symptoms reduced more substantially than less common ones. Of course, many of these more common symptoms (such as chest infections and sore throats) will be attributable to causes other than cancer, such as viral infections, and the figures should be interpreted in this context. Social distancing and lockdown has resulted in less infection overall as demonstrated by the Royal College of General Practitioners research and surveillance centre data, which shows a marked reduction in weekly reported incidence of asthma, intestinal infectious diseases, and upper and acute respiratory tract infections from week 12 of 2020.[24] Nonetheless, the reductions are still concerning, especially for older patients. A 2020 survey from Cancer Research UK suggested GPs are particularly worried about older people not consulting with potential cancer symptoms compared to before the pandemic.[5] Analysis of the national cancer registry in the Netherlands shows reduction in cancer diagnosis at the start of the pandemic was particularly pronounced in patients aged 80 years or over.[9] The Netherlands operates a similar GP gatekeeper model to the UK[25], and the authors of this Dutch paper suggested that the move to remote GP consulting combined with patients delaying consultation about potential non-specific cancer symptoms may have contributed to the reduction in diagnoses.[9] In the current study we show that this suggestion is supported for the set of UK practices we analysed. Although we did not observe a greater reduction in symptom reporting in patients aged 80 or over, potential symptoms in over 80s are more likely to result in a cancer diagnosis; therefore, the fact that reductions were similar across all age groups, rather than less of a reduction seen in older age groups, is of concern.

Implications for policy and practice

Combined with our previous work,[4] the Cancer Research UK report,[5] and reductions in cancer diagnoses[6 7] these findings suggest that patients are less likely to report potential cancer indicators than before the COVID-19 pandemic, particularly for more common symptoms such as fever and coughs but also for alarm symptoms. In the context of repeated lockdowns, it is therefore extremely important that the general public are advised that they should still consult with primary care if they have persistent symptoms that might indicate cancer. Further, GPs and nurses should be encouraged to ask more probing questions during remote consulting, as they may miss symptoms which may have previously been picked up from non-verbal cues and possibly a more open discussion face-to-face.

Conclusion

The proportion of patients consulting with primary care, as well as those reporting potential cancer indicators, reduced during the first-wave of the COVID-19 pandemic. While there has been an increased rate of potential cancer indicators reported in remote consultations compared to last year, there are still lower rates in remote GP consultations than in face-to-face, suggesting remote consulting may be part of the reason for the reduction in reporting of potential cancer indicators.

For peer review only - http://bmjopen.bmj.com/site/about/guidelines.xhtml

UOB Confidential & Sensitive

Acknowledgements: The authors would like to thank all the participants in this study, Bristol North Somerset and South Gloucestershire Clinical Commissioning Group, One Care for providing the data extract, the NIHR Clinical Research Network for adopting the study on the NIHR portfolio, and the NIHR SPCR for funding the research. We would also like to thank Willie Hamilton for his work on developing the potential cancer indicator lists (alongside author Sarah Price).

Funding: This study was funded by the National Institute for Health Research (NIHR) School for Primary Care Research. Additional funding for staff time was provided by the Applied Research Collaboration West (ARC West) at University Hospitals Bristol and Weston NHS Foundation Trust and One Care. Professor Salisbury is a National Institute for Health Research (NIHR) Senior Investigator. SP is funded by the National Institute for Health Research (NIHR) Policy Research Programme via the Policy Research Unit in Cancer Awareness, Screening and Early Diagnosis, PR-PRU-1217-21601. The funder had no role in the study design, collection and analysis of data, or the writing of the manuscript. The views expressed are those of the authors and not necessarily those of, the NIHR or the Department of Health and Social Care. TP is supported by the Integrative Epidemiology Unit, which receives funding from the UK Medical Research Council and the University of Bristol (MC_UU_00011/1 and MC_UU_00011/3)

Ethical approval: This study received ethical approval from the University of Bristol Faculty of Health Sciences Research Ethics Committee (ID 103166), and Health Research Authority approval (IRAS project ID 282541; REC reference 20/HRA/2070). The study was sponsored by the University of Bristol.

Competing interests: The authors declare that they have no competing interests

Data sharing: Under the Data Transfer Standard Operating Procedure agreed with practices, the patient data for this study will not be shared outside the research team. The codes lists used in the analysis which identify symptoms, signs, test results or diagnoses which could potentially indicate cancer can be made available upon reasonable request.

Transparency statement: All authors affirm that the manuscript is an honest, accurate, and transparent account of the study being reported, that no important aspects of the study have been omitted, and that any discrepancies from the study as originally planned have been explained.

Dissemination declaration: Results will not be disseminated to study participants as they were not directly involved or consented into the study and it would therefore not be appropriate.

Contributions of authors: LS, MM, RD, JH, and CS contributed to the conception and design of the study, SP provided the potential cancer indicator code lists and interpretation of these, RL extracted the data, MM led the main study, and LS performed the analysis and drafted the manuscript. MM updated the paper following reviewer comments and TP performed analysis for this. All authors contributed to the organisation and conduct of the study, the interpretation of study data and results, and critiqued the manuscript for important intellectual content.

For peer review only - http://bmjopen.bmj.com/site/about/guidelines.xhtml

UOB Confidential & Sensitive

References

1. Organisation WH. WHO Director-General's opening remarks at the media briefing on COVID-19 - 11 March 2020, 2020.
2. Care DoHaS. Coronavirus: stay at home, protect the NHS, save lives - web version 2020 [Available from: https://www.gov.uk/government/publications/coronavirus-covid-19-information-leaflet/coronavirus-stay-at-home-protect-the-nhs-save-lives-web-version.
3. Andrea D. GPs urged to go digital to prevent spread of coronavirus 2020 [updated 2020/10/03/. Available from: https://www.digitalhealth.net/2020/03/gps-urged-to-go-digital-to-prevent-spread-of-coronavirus/.
4. Murphy M, Scott LJ, Salisbury C, et al. The implementation of remote consulting in UK primary care following the COVID-19 pandemic: a mixed-methods longitudinal study. *Research Square- PRE-PRINT* 2020 doi: 10.21203/rs.3.rs-94227/v1
5. Cancer Research UK. GPs say elderly are not seeking help for potential cancer symptoms, 2020.
6. Andrew TW, Alrawi M, Lovat P. Reduction in skin cancer diagnoses in the UK during the COVID-19 pandemic. *Clin Exp Dermatol* 2021;46(1):145-46. doi: 10.1111/ced.14411 [published Online First: 2020/08/06]
7. Morris EJA, Goldacre R, Spata E, et al. Impact of the COVID-19 pandemic on the detection and management of colorectal cancer in England: a population-based study. *Lancet Gastroenterol Hepatol* 2021 doi: 10.1016/S2468-1253(21)00005-4 [published Online First: 2021/01/18]
8. Skovlund CW, Friis S, Dehlendorff C, et al. Hidden morbidities: drop in cancer diagnoses during the COVID-19 pandemic in Denmark. *Acta Oncol* 2021;60(1):20-23. doi: 10.1080/0284186X.2020.1858235 [published Online First: 2020/12/09]
9. Dinmohamed AG, Visser O, Verhoeven RHA, et al. Fewer cancer diagnoses during the COVID-19 epidemic in the Netherlands. *Lancet Oncol* 2020;21(6):750-51. doi: 10.1016/S1470-2045(20)30265-5 [published Online First: 2020/05/04]
10. Jones D, Neal RD, Duffy SRG, et al. Impact of the COVID-19 pandemic on the symptomatic diagnosis of cancer: the view from primary care. *Lancet Oncol* 2020;21(6):748-50. doi: 10.1016/S1470-2045(20)30242-4 [published Online First: 2020/05/04]
11. Richards M, Anderson M, Carter P, et al. The impact of the COVID-19 pandemic on cancer care. *Nat Cancer* 2020:1-3. doi: 10.1038/s43018-020-0074-y [published Online First: 2020/08/25]
12. Neal RD, Tharmanathan P, France B, et al. Is increased time to diagnosis and treatment in symptomatic cancer associated with poorer outcomes? Systematic review. *Br J Cancer* 2015;112 Suppl 1:S92-107. doi: 10.1038/bjc.2015.48 [published Online First: 2015/03/04]
13. Maringe C, Spicer J, Morris M, et al. The impact of the COVID-19 pandemic on cancer deaths due to delays in diagnosis in England, UK: a national, population-based, modelling study. *Lancet Oncol* 2020;21(8):1023-34. doi: 10.1016/S1470-2045(20)30388-0 [published Online First: 2020/07/24]
14. Price S, Spencer A, Zhang X, et al. Trends in time to cancer diagnosis around the period of changing national guidance on referral of symptomatic patients: A serial cross-sectional study using UK electronic healthcare records from 2006-17. *Cancer Epidemiol* 2020;69:101805. doi: 10.1016/j.canep.2020.101805 [published Online First: 2020/09/13]
15. Watson J, Nicholson BD, Hamilton W, et al. Identifying clinical features in primary care electronic health record studies: methods for codelist development. *BMJ Open* 2017;7(11):e019637. doi: 10.1136/bmjopen-2017-019637 [published Online First: 2017/11/25]
16. National Institute for Health and Care Excellence. Suspected cancer: recognition and referral. NICE guideline [NG12], 2020.
17. Clinical Practice Research Datalink. CPRD; UK data driving real-world evidence [Available from: www.cprd.com2020.

For peer review only - http://bmjopen.bmj.com/site/about/guidelines.xhtml

UOB Confidential & Sensitive

18. NHS Digital. Quality and Outcomes Framework (QOF) business rules v42 2019-2020 baseline release, 2019/2020.
19. NHS Digital. Patients Registered at a GP Practice 2019-2020 [Available from: https://digital.nhs.uk/data-and-information/publications/statistical/patients-registered-at-a-gp-practice2020.
20. GOV.UK. Population of England and Wales: UK population by ethnicity 2018 [Available from: www.ethnicity-facts-figures.service.gov.uk/uk-population-by-ethnicity/national-and-regional-populations/population-of-england-and-wales/latest#by-ethnicity.
21. Price SJ, Stapley SA, Shephard E, et al. Is omission of free text records a possible source of data loss and bias in Clinical Practice Research Datalink studies? A case-control study. *BMJ Open* 2016;6(5):e011664. doi: 10.1136/bmjopen-2016-011664 [published Online First: 2016/05/15]
22. Williams R, Jenkins DA, Ashcroft DM, et al. Diagnosis of physical and mental health conditions in primary care during the COVID-19 pandemic: a retrospective cohort study. *Lancet Public Health* 2020;5(10):e543-e50. doi: 10.1016/S2468-2667(20)30201-2 [published Online First: 2020/09/27]
23. Hammersley V, Donaghy E, Parker R, et al. Comparing the content and quality of video, telephone, and face-to-face consultations: a non-randomised, quasi-experimental, exploratory study in UK primary care. *Br J Gen Pract* 2019;69(686):e595-e604. doi: 10.3399/bjgp19X704573 [published Online First: 2019/07/03]
24. Royal College of General Practitioners. Research and Surveillance Centre workload observatory 2020 [Available from: https://clininf.eu/index.php/rcgprscworkloadobservatory/.
25. Faber MJ, Burgers JS, Westert GP. A sustainable primary care system: lessons from the Netherlands. *J Ambul Care Manage* 2012;35(3):174-81. doi: 10.1097/JAC.0b013e31823e83a4 [published Online First: 2012/06/07]

For peer review only

For peer review only - http://bmjopen.bmj.com/site/about/guidelines.xhtml

UOB Confidential & Sensitive

Table 1: Characteristics of registered and consulting patients in participating practices

	All patients registered in July 2020 (N=126,466) *		Patients consulting in April-July 2019 (N=69,254)		Patients consulting in April-July 2020 (N=56,314)	
	n	%	n	%	n	%
Age						
50-59 years	44,614	35.3%	20,830	30.1%	16,711	29.7%
60-69 years	33,338	26.4%	17,765	25.7%	14,252	25.3%
70-79 years	27,536	21.8%	17,512	25.3%	14,066	25.0%
80+ years	20,978	16.6%	13,147	19.0%	11,285	20.0%
Gender						
Male	61,798	48.9%	30,949	44.7%	25,129	44.6%
Female	64,668	51.1%	38,305	55.3%	31,185	55.4%
Index of multiple deprivation quintile						
1 (most deprived)	19,392	15.4%	10,877	15.8%	9,355	16.7%
2	15,907	12.7%	8,792	12.8%	7,403	13.2%
3	18,665	14.9%	10,197	14.8%	8,151	14.5%
4	28,169	22.4%	15,174	22.0%	12,236	21.8%
5 (least deprived)	43,500	34.6%	23,861	34.6%	18,888	33.7%
Missing	*833*		*353*		*281*	
Ethnicity**						
White	91,401	93.2%	52,262	93.3%	42,512	93.3%
Asian	2,507	2.6%	1,443	2.6%	1,161	2.5%
Black	2,963	3.0%	1,655	3.0%	1,374	3.0%
Mixed	1,000	1.0%	532	0.9%	425	0.9%
Other	227	0.2%	111	0.2%	89	0.2%
Missing	*28,368*		*13,251*		*10,753*	
Mental health status						
No MH conditions	111,143	87.9%	57,923	83.6%	45,885	81.5%
1+ MH condition	15,323	12.1%	11,331	16.4%	10,429	18.5%
Shielding status						
Not advised to shield	116,518	92.1%	61,417	88.7%	48,305	85.8%
Advised to shield	9,948	7.9%	7,837	11.3%	8,009	14.2%

* The corresponding number of patients registered in 2019 was 123,947. As we only had patient characteristic data for the 21 participating practices for patients registered on 1st July 2020, we imputed the 2019 list sizes using NHS digital historical list sizes.

** White includes British, white British, mixed British, and other white background. Black includes African, Caribbean, Somali and black British. Asian includes Indian, British Indian, Pakistani, Chinese, and other Asian background. Mixed includes white and black Caribbean, white and Asian, white and African, other mixed white, and other mixed background. Other includes, Turkish, Arab, Iranian, other ethnic non-mixed, and any other group. See supplemental file 1 for further details.

For peer review only - http://bmjopen.bmj.com/site/about/guidelines.xhtml

UOB Confidential & Sensitive

Table 2: Patients with any potential cancer indicator reported in April-July 2020 compared to 2019

	April-July 2019 (n=123,947)		April-July 2020 (n=126,466)		Change in proportion of patients with potential cancer indicators (2020 vs. 2019)		
	n	% [a]	n	% [a]	IRR	95% CI	p-value
Overall	21,252	17%	13,829	11%	0.64	0.62 to 0.67	<0.001
By Age category							0.136[b]
50-59 years	6,217	14%	4,083	9%	0.65	0.61 to 0.69	<0.001
60-69 years	5,398	17%	3,368	10%	0.61	0.58 to 0.65	<0.001
70-79 years	5,457	20%	3,449	13%	0.62	0.59 to 0.67	<0.001
80+ years	4,180	20%	2,929	14%	0.68	0.64 to 0.73	<0.001
By Sex							0.467[b]
Male	9,202	15%	5,905	10%	0.63	0.60 to 0.67	<0.001
Female	12,050	19%	7,924	12%	0.65	0.62 to 0.68	<0.001
By Ethnicity [c]							0.471[b]
White	15,910	18%	10,340	11%	0.64	0.61 to 0.68	<0.001
Asian	590	24%	357	14%	0.58	0.50 to 0.69	<0.001
Black	710	24%	497	17%	0.68	0.58 to 0.80	<0.001
Mixed	193	20%	106	11%	0.55	0.42 to 0.70	<0.001
Other	40	18%	25	11%	0.60	0.36 to 1.00	0.050
By index of multiple deprivation quintile							0.585[b]
1 (most deprived)	3,916	21%	2,662	14%	0.67	0.63 to 0.72	<0.001
2	2,978	19%	1,981	12%	0.64	0.60 to 0.69	<0.001
3	3,158	17%	2,052	11%	0.63	0.59 to 0.68	<0.001
4	4,607	17%	2,999	11%	0.63	0.59 to 0.67	<0.001
5 (least deprived)	6,469	15%	4,053	9%	0.62	0.58 to 0.67	<0.001
By Mental health status							<0.001[b]
No MH conditions	17,338	16%	10,924	10%	0.62	0.60 to 0.64	<0.001
1+ MH condition	3,914	26%	2,905	19%	0.73	0.69 to 0.77	<0.001
By Shielding status							0.099[b]
Not advised to shield	17,956	16%	11,564	10%	0.63	0.61 to 0.66	<0.001
Advised to shield	3,296	34%	2,265	23%	0.68	0.63 to 0.72	<0.001

IRR=incidence rate ratio. CI=Confidence interval. IRRs and corresponding 95% CIs and p-values from negative binomial models.

[a] Percentages are calculated out of all patients in each category.

[b] P-values presented on the blue rows are the p-values for the interaction between year and the given characteristic; incidence rate ratios only presented by each level of a characteristic if this $p<0.05$.

[c] White includes British, white British, mixed British, and other white background. Black includes African, Caribbean, Somali and black British. Asian includes Indian, British Indian, Pakistani, Chinese, and other Asian background. Mixed includes white and black Caribbean, white and Asian, white and African, other mixed white, and other mixed background. Other includes, Turkish, Arab, Iranian, other ethnic non-mixed, and any other group. See supplemental file 1 for further details.

For peer review only - http://bmjopen.bmj.com/site/about/guidelines.xhtml

UOB Confidential & Sensitive

For peer review only

Table 3: Consultations (per 1000 patients) with potential cancer indicators reported in April-July 2020 compared to 2019

	April-July 2019		April-July 2020		Change in rates of consultations with potential cancer indicators (2020 vs. 2019)		
GP CONSULTATIONS	n	%[a]	n	%[a]	IRR	95% CI	p-value
Overall	236.5	19%	176.4	15%	0.79	0.74 to 0.84	<0.001
By consultation provision							<0.001 [b]
Face-to-face	187.8	21%	25.4	17%	0.84	0.76 to 0.92	<0.001
Remote	48.7	13%	151.0	14%	1.17	1.07 to 1.29	0.001
NURSE/PARAMEDIC CONSULTATIONS	n	%	n	%	IRR	95% CI	p-value
Overall	53.6	9%	31.9	7%	0.69	0.56 to 0.85	<0.001
By consultation provision							<0.001 [b]
Face-to-face	50.2	10%	15.7	6%	0.61	0.44 to 0.83	0.002
Remote	3.4	7%	16.1	9%	1.60	1.10 to 2.33	0.014

IRR=incidence rate ratio. CI=Confidence interval. IRRs and corresponding 95% CIs and p-values from negative binomial models.

[a] Percentages are calculated out of all consultations in each category.

[b] P-values presented on the blue rows are the p-values for the interaction between year and consultation provision.

peer review only

For peer review only - http://bmjopen.bmj.com/site/about/guidelines.xhtml

UOB Confidential & Sensitive

Table 4: Patients with individual potential cancer indicator reported in April-July 2020 compared to 2019

	April-July 2019 (n=123,947)		April-July 2020 (n=126,466)			95% CI (Bold indicates p<0.0008)
	n	%	n	%	IRR	
Any potential cancer indicator	21,252	17.15%	13,829	10.93%	0.64	**0.62 - 0.65**
**Cancer alarm symptoms (see *)**	1,801	1.45%	1,344	1.06%	0.73	**0.68 - 0.79**
Most commonly reported indicators (≥0.5% of patients in 2019)						
Any commonly reported indicator	*16,632*	*13.42%*	*10,054*	*7.95%*	*0.59*	**0.58 - 0.61**
Chest infection	1,427	1.15%	370	0.29%	0.25	**0.23 - 0.29**
Fever	1,216	0.98%	443	0.35%	0.36	**0.32 - 0.40**
Cough	3,125	2.52%	1,397	1.10%	0.44	**0.41 - 0.47**
Fatigue	1,284	1.04%	655	0.52%	0.50	**0.45 - 0.55**
Urinary tract infection	760	0.61%	394	0.31%	0.51	**0.45 - 0.57**
Pain in shoulder	1,307	1.05%	785	0.62%	0.59	**0.54 - 0.64**
Proteinuria	697	0.56%	437	0.35%	0.61	**0.54 - 0.69**
Non-visible haematuria	979	0.79%	616	0.49%	0.62	**0.56 - 0.68**
Pain in abdomen	1,908	1.54%	1,301	1.03%	0.67	**0.62 - 0.72**
Haematuria*	897	0.72%	612	0.48%	0.67	**0.60 - 0.74**
Shortness of breath	2,382	1.92%	1,650	1.30%	0.68	**0.64 - 0.72**
Pain in chest	1,180	0.95%	830	0.66%	0.69	**0.63 - 0.75**
Back pain/backache	2,659	2.15%	1,887	1.49%	0.70	**0.66 - 0.74**
Lower urinary tract symptoms	1,402	1.13%	1,027	0.81%	0.72	**0.66 - 0.78**
Compressed trachea	864	0.70%	638	0.50%	0.72	**0.65 - 0.80**
Diarrhoea	860	0.69%	645	0.51%	0.74	**0.66 - 0.82**
Less commonly reported indicators (0.1%-<0.5% of patients in 2019)						
Any less commonly reported indicator	*6,585*	*5.31%*	*5,008*	*3.96%*	*0.75*	**0.72 - 0.77**
Erectile dysfunction	363	0.29%	148	0.12%	0.40	**0.33 - 0.49**
Irritable Bowel Syndrome	194	0.16%	97	0.08%	0.49	**0.38 - 0.63**
Gynae abnormalities (e.g. menstrual disorders and unusual bleeding)	190	0.15%	99	0.08%	0.51	**0.40 - 0.65**
Pain in throat (e.g. tonsilitis)	201	0.16%	109	0.09%	0.53	**0.42 - 0.67**
Iron deficiency anaemias	477	0.38%	273	0.22%	0.56	**0.48 - 0.65**
Change in bowel habit	281	0.23%	161	0.13%	0.56	**0.46 - 0.68**
Pain in breast	170	0.14%	99	0.08%	0.57	**0.44 - 0.74**
Bruising	208	0.17%	126	0.10%	0.59	**0.47 - 0.74**
Prostate specific antigen raised/abnormal	173	0.14%	121	0.10%	0.69	**0.54 - 0.87**
Postmenopausal bleeding*	144	0.12%	101	0.08%	0.69	0.53 - 0.89
Pain in pelvis	388	0.31%	280	0.22%	0.71	**0.60 - 0.83**
Pruritus	356	0.29%	259	0.20%	0.71	**0.61 - 0.84**
Vomiting	245	0.20%	180	0.14%	0.72	**0.59 - 0.88**
Dyspepsia	590	0.48%	450	0.36%	0.75	**0.66 - 0.85**
Distension of abdomen	142	0.11%	111	0.09%	0.77	0.59 - 0.99
Reflux	560	0.45%	439	0.35%	0.77	**0.68 - 0.87**
Lump in breast*	208	0.17%	170	0.13%	0.80	0.65 - 0.99
Recurring infections	366	0.30%	304	0.24%	0.81	0.70 - 0.95
Rectal bleeding*	357	0.29%	303	0.24%	0.83	0.71 - 0.97
Dysuria	196	0.16%	169	0.13%	0.85	0.68 - 1.04

For peer review only - http://bmjopen.bmj.com/site/about/guidelines.xhtml

UOB Confidential & Sensitive

	April-July 2019 (n=123,947)		April-July 2020 (n=126,466)			95% CI (Bold indicates p<0.0008)
	n	%	n	%	IRR	
Vaginal discharge	153	0.12%	134	0.11%	0.86	0.68 - 1.09
Dysphagia*	171	0.14%	151	0.12%	0.87	0.69 - 1.08
Lump (excluding breast)	402	0.32%	375	0.30%	0.91	0.79 - 1.06
Nausea	147	0.12%	150	0.12%	1.00	0.79 - 1.26
Weight loss	218	0.18%	239	0.19%	1.07	0.89 - 1.30
Constipation	545	0.44%	655	0.52%	1.18	1.05 - 1.32
Rarely reported indicators (0.02- <0.1% of patients in 2019)						
Any rarely reported indicator	*909*	*0.73%*	*702*	*0.56%*	*0.76*	**0.68 - 0.84**
Limp/ gait abnormalities	25	0.02%	10	0.01%	0.39	0.17 - 0.85
Night sweats	75	0.06%	33	0.03%	0.43	**0.28 - 0.66**
Lymphadenopathy	86	0.07%	38	0.03%	0.43	**0.29 - 0.64**
Haemoptysis*	62	0.05%	34	0.03%	0.54	0.34 - 0.83
Abnormal chest x-ray	28	0.02%	17	0.01%	0.60	0.31 - 1.13
Hoarseness of voice	101	0.08%	65	0.05%	0.63	0.45 - 0.87
Pain in testis	71	0.06%	46	0.04%	0.63	0.43 - 0.93
Pain in bones	118	0.10%	86	0.07%	0.71	0.53 - 0.95
Possible bone and soft tissue sarcoma symptoms (e.g. abnormal x-ray)	30	0.02%	22	0.02%	0.72	0.40 - 1.29
Chest/breathing abnormalities	26	0.02%	20	0.02%	0.75	0.40 - 1.40
Mass in neck	115	0.09%	89	0.07%	0.76	0.57 - 1.01
Mass in testis	102	0.08%	79	0.06%	0.76	0.56 - 1.03
Superior vena cava syndrome	39	0.03%	38	0.03%	0.95	0.59 - 1.53
Testis swelling/abnormalities	31	0.03%	32	0.03%	1.01	0.60 - 1.71
Appetite loss	49	0.04%	76	0.06%	1.52	1.05 - 2.22
Mass in abdomen	42	0.03%	79	0.06%	1.84	**1.25 - 2.75**
Very rarely reported indicators (<0.02% of patients in 2019)						
*Any very rarely reported indicator***	*141*	*0.11%*	*108*	*0.09%*	*0.75*	0.58 - 0.97

IRR=Incidence rate ratio. Bold face indicates a change over time based on 95%CI.

Note. Indicators are ordered from largest to smallest reduction (by IRR) within each commonality grouping. These figures only include indicators coded in routinely collected records using SNOMED codes. In some cases, GPs/nurses may enter symptoms in free text, and/or only code their diagnosis, which may lead to under-reporting here; this under-recording is likely to be similar in both years.

* "Alarm" symptoms, which warrant investigation in their own right in NICE suspected-cancer guidelines, because of their predictive power for cancer: visible haematuria, rectal bleeding, haematemesis, haemoptysis, jaundice, breast lump, postmenopausal bleeding in women aged 55 and over, and dysphagia

** Very rare indicators affecting <0.02% of patients (25 individuals) in April-July 2019 clubbing of fingers, ulceration of vulva, prostate abnormalities on digital rectal exam, nipple retraction, skin lesion, mass in thyroid, jaundice, spinal cord compression, mass in pelvis, faecal occult blood test, abnormal skin on breast, nipple discharge, haematemesis and 12 additional indicators with incidence <5 patients in April-July 2019.

For peer review only - http://bmjopen.bmj.com/site/about/guidelines.xhtml

UOB Confidential & Sensitive

Figure 1:
Title: Proportion of patients consulting and proportion consulting with potential cancer indicators

Figure 2:
Title: Number of consultations per 1000 patients and proportion of those with potential cancer indicators

For peer review only

For peer review only - http://bmjopen.bmj.com/site/about/guidelines.xhtml

UOB Confidential & Sensitive

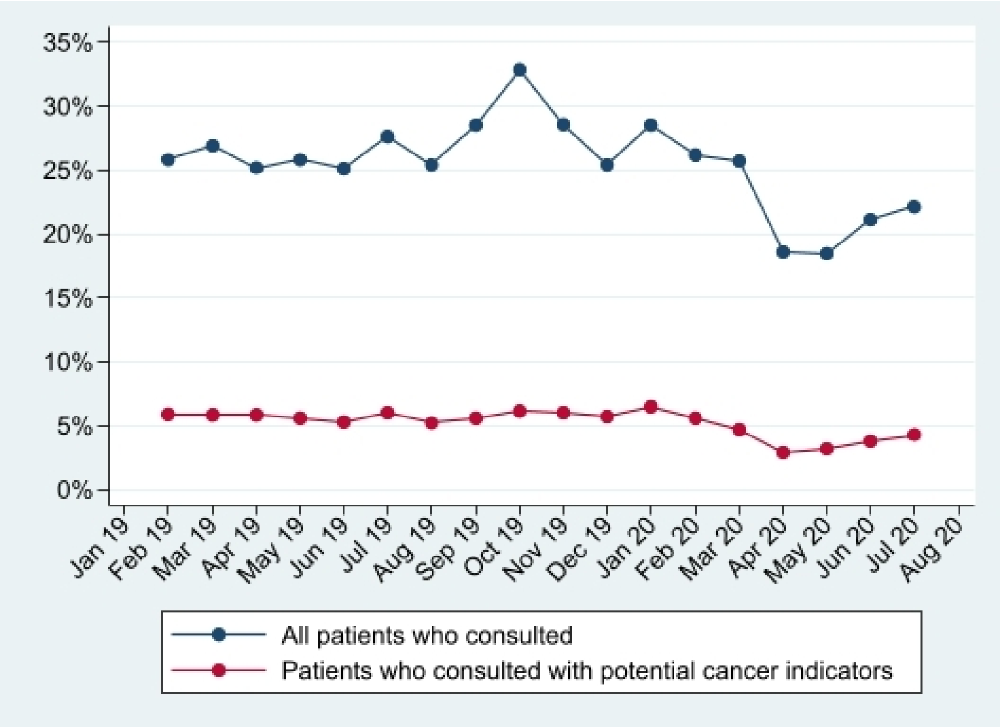

Figure 1: Proportion of patients consulting and proportion consulting with potential cancer indicators

139x101mm (96 x 96 DPI)

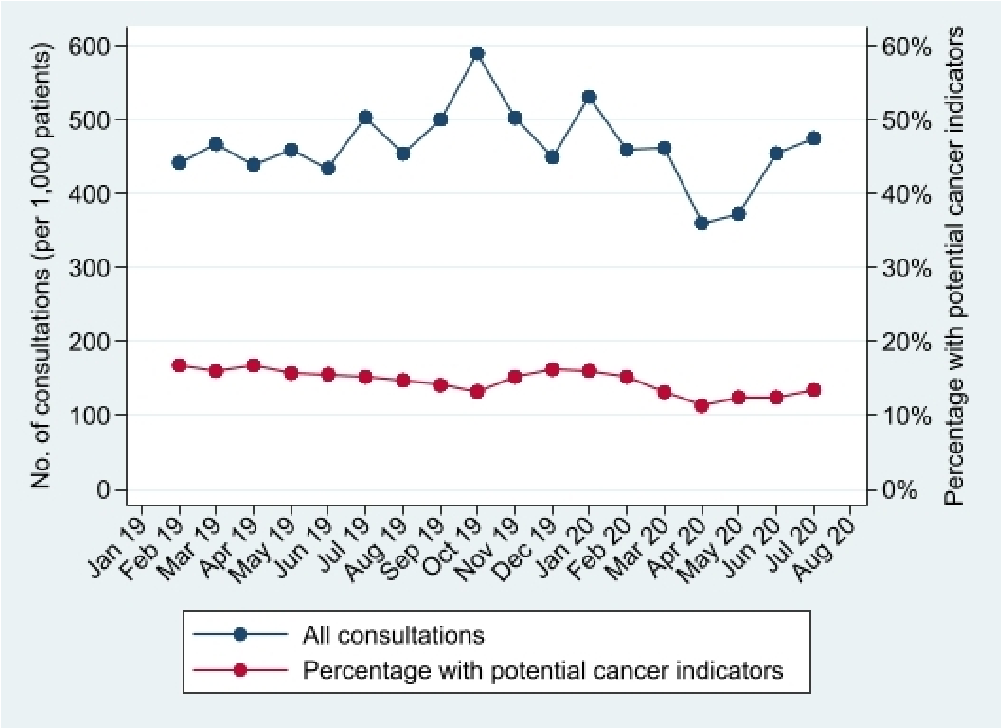

Figure 2: Number of consultations per '000 patients and proportion of those with potential cancer indicators

139x101mm (96 x 96 DPI)

Supplementary File 1: Data Rules

1. Data extraction and analysis

- ⇨ Data on patient characteristics, consultations and clinical codes for 21 practices were extracted by One Care.
- ⇨ We only extracted consultations added by clinicians. Administrator added consultations (e.g. e-consultations and documents uploaded) were not extracted.
- ⇨ Patient characteristics were extracted for all patients registered in July 2020.
- ⇨ Consultations were extracted for all patients registered in July 2020.
- ⇨ To calculate consultations per 1000 patients, we used the July 2020 patient list extracted by One Care as the denominator for the April-July 2020 consultations. For the April-July 2019 data, we adjusted this list size based on practice list size data (total list size of patients aged 50+ years for all 21 included practices) published by NHS digital. We calculated the quotient of July 2019/July 2020 list size, and used this as a multiplier for the denominator. We used this same method to individually calculate the offset for each practice for the negative binomial models. For the figures presenting data by month, we again used this same method but using monthly list sizes (for patients aged 50+ years) on NHS digital to calculate the multiplier for the denominator.

2. Consultation Type

We applied the following rules to identify consultation type:

Face-to-face: If

consultation category= "gp surgery" OR "face to face consultation" OR "face to face consultation with relative/carer" OR "emergency appointment" OR "extended hours consultation" OR "same day appointment" OR "urgent consultation".

Home: If
consultation category= "children's home visit note" OR "home visit note" OR "night visit note" OR "nursing home visit note" OR "residential home visit note" OR "twilight visit note"
OR
Clinical code text contains the words "home visit".

Telephone: If
consultation category contains the words "telephone call" OR "telephone consultation" OR "enterprise consultation"[1]
OR
appointment type contains the words "telephone" OR "phonecall" OR "phone call" OR "telcon"
OR
appointment slot status= "telephone – complete"
OR

---

[1] These are consultations carried out in a GP surgery which is not the patients own surgery. We identified these as telephone consultation as we do not know anything more about the medium of these consultations and telephone was most likely. If clinical codes for video consultation were identified, this was coded to video instead, in accordance with the data rules shown in the following page.

For peer review only

Clinical code text contains the word “telephone” OR “telephone and/or video” OR “telephone/video” OR “telephone/ video” OR “telephone / video”

Video: If
consultation category= “consultation via video conference"
OR
Clinical code text= assessed by video consult" OR "consultation via video conference" OR "video consult"
E-consultation[2]: If
consultation category= “online communication” OR “consultation via multimedia” OR contains the word “e-mail”
OR
appointment type contains the words “e consult” OR “econsult” OR “e:consult”
OR
Clinical codes text contains the words “econsult” OR “e-consult” OR “e:consult” OR “e consult” OR “consultation via multimedia” OR “alert received from telehealth monitoring system”

We used the following rank order for determining consultation type: **Video, Telephone, E-consult, Home, Face-to-face** (E.g. if a patient has consultation type as “GP surgery” but has a clinical code for "video consult" then this is coded as video.)

Consultations were excluded as follows:

- Non consultation activity (e.g. where the consultation category was “administration note”, “inbound document”, and “scanned document”).
- Records which were appearing as consultations but were actually SMS messages. Identified as: *Consultation type*= ”Face-to-face” AND *consultation category*=”gp surgery” AND the consultation has a maximum of 3 clinical codes associated to it, and one is “patient mobile telephone number” or “patient telephone number”, and one is “sms (short message service) text message sent to patient” or “sms text message sent to patient” or “sms text sent to patient”
- Consultations which the patient did not attend, or did not answer the phone: Identified by (*Clinical code text* contains the words “did not attend” OR “failed encounter” OR “dna” AND *clinical code text* does not equal “dna studies” OR “dnacpr”) OR (*appointment slot status* = “telephone - not in” OR “dna” OR “walked out”).

3. Staff type

Codes were mapped for staff type as follows:

GP: Assistant GP, Associate practitioner - general practitioner, General medical practitioner, GP registrar, Locum GP, Salaried general practitioner, Sessional GP

[2] Because we only included consultations added by clinicians, not administrators, these only include e-consultations which clinicians added to the patient record. E-consultations added by administrators which covert to a telephone call (added by a GP) or dealt with by a task are not included.

For peer review only

Nurse/Paramedic: Advanced practitioner, Associate practitioner – nurse, Community nurse, Community practitioner, Enrolled nurse, Modern matron, Nurse consultant, Nurse manager, Paramedic, Paramedic specialist practitioner, Sister/charge nurse, Specialist practitioner, Staff nurse.

An Allied Professional Staff Type was also created. However, as the numbers of consultations were very small, this was not used in our analysis.

4. Ethnicity

Codes were mapped for ethnicity as follows:

- ⇨ **White**: 44 different codes. Over 90% of patients were covered by the codes "White British", "British or mixed British" or "other white background."
- ⇨ **Black/African/Caribbean/Black British**: 36 different codes. Over 90% were coded as "African", "Caribbean", "black African", "black Caribbean", "Somali", or "black British".
- ⇨ **Asian/Asian British**: 49 different codes. 90% of patients were covered by the codes "Indian or British Indian", "other Asian background", "Pakistani or British Pakistani", "Chinese", "Bangladeshi or British Bangladeshi", "Indian" or "Pakistani".
- ⇨ **Mixed/Multiple ethnic groups**: 27 different codes: 90% covered by "white and black Caribbean", "other mixed background", "white and Asian", "white and black African", "other mixed white", "black and white".
- ⇨ **Other**: 40 codes including: "any other group", "Turkish", "Arab", "other ethnic non-mixed (nmo)", "Iranian", "gypsy/Romany", "Kurdish", "Latin American", "Albanian", "other European (nmo)".

For peer review only

STROBE Statement—Checklist of items that should be included in reports of ***cohort studies***

	Item No	Recommendation	Page
**Title and abstract**	1	(*a*) Indicate the study's design with a commonly used term in the title or the abstract	2
		(*b*) Provide in the abstract an informative and balanced summary of what was done and what was found	2
**Introduction**			
Background/rationale	2	Explain the scientific background and rationale for the investigation being reported	4
Objectives	3	State specific objectives, including any prespecified hypotheses	4
**Methods**			
Study design	4	Present key elements of study design early in the paper	4-6
Setting	5	Describe the setting, locations, and relevant dates, including periods of recruitment, exposure, follow-up, and data collection	4
Participants	6	(*a*) Give the eligibility criteria, and the sources and methods of selection of participants. Describe methods of follow-up	4
		(*b*) For matched studies, give matching criteria and number of exposed and unexposed	-
Variables	7	Clearly define all outcomes, exposures, predictors, potential confounders, and effect modifiers. Give diagnostic criteria, if applicable	4-6
Data sources/ measurement	8*	For each variable of interest, give sources of data and details of methods of assessment (measurement). Describe comparability of assessment methods if there is more than one group	4-6
Bias	9	Describe any efforts to address potential sources of bias	5
Study size	10	Explain how the study size was arrived at	-
Quantitative variables	11	Explain how quantitative variables were handled in the analyses. If applicable, describe which groupings were chosen and why	5-6
Statistical methods	12	(*a*) Describe all statistical methods, including those used to control for confounding	5-6
		(*b*) Describe any methods used to examine subgroups and interactions	5-6
		(*c*) Explain how missing data were addressed	-
		(*d*) If applicable, explain how loss to follow-up was addressed	-
		(*e*) Describe any sensitivity analyses	-
**Results**			
Participants	13*	(a) Report numbers of individuals at each stage of study—eg numbers potentially eligible, examined for eligibility, confirmed eligible, included in the study, completing follow-up, and analysed	6-7
		(b) Give reasons for non-participation at each stage	-
		(c) Consider use of a flow diagram	-
Descriptive data	14*	(a) Give characteristics of study participants (eg demographic, clinical, social) and information on exposures and potential confounders	6, Table 1
		(b) Indicate number of participants with missing data for each variable of interest	Table 1
		(c) Summarise follow-up time (eg, average and total amount)	6-7
Outcome data	15*	Report numbers of outcome events or summary measures over time	6-7

For peer review only

For peer review only - http://bmjopen.bmj.com/site/about/guidelines.xhtml

Main results	16	(*a*) Give unadjusted estimates and, if applicable, confounder-adjusted estimates and their precision (eg, 95% confidence interval). Make clear which confounders were adjusted for and why they were included	6-7
		(*b*) Report category boundaries when continuous variables were categorized	6-7 Tables 1,2,3
		(*c*) If relevant, consider translating estimates of relative risk into absolute risk for a meaningful time period	-
Other analyses	17	Report other analyses done—eg analyses of subgroups and interactions, and sensitivity analyses	6-7
**Discussion**			
Key results	18	Summarise key results with reference to study objectives	7
Limitations	19	Discuss limitations of the study, taking into account sources of potential bias or imprecision. Discuss both direction and magnitude of any potential bias	8
Interpretation	20	Give a cautious overall interpretation of results considering objectives, limitations, multiplicity of analyses, results from similar studies, and other relevant evidence	7-9
Generalisability	21	Discuss the generalisability (external validity) of the study results	8
**Other information**			
Funding	22	Give the source of funding and the role of the funders for the present study and, if applicable, for the original study on which the present article is based	10

*Give information separately for exposed and unexposed groups.

Note: An Explanation and Elaboration article discusses each checklist item and gives methodological background and published examples of transparent reporting. The STROBE checklist is best used in conjunction with this article (freely available on the Web sites of PLoS Medicine at http://www.plosmedicine.org/, Annals of Internal Medicine at http://www.annals.org/, and Epidemiology at http://www.epidem.com/). Information on the STROBE Initiative is available at http://www.strobe-statement.org.

For peer review only

For peer review only - http://bmjopen.bmj.com/site/about/guidelines.xhtml
